# Eliciting a single amino acid change by vaccination generates antibody protection against group 1 and group 2 influenza A viruses

**DOI:** 10.1016/j.immuni.2024.03.022

**Published:** 2024-04-25

**Authors:** Rashmi Ray, Faez Amokrane Nait Mohamed, Daniel P. Maurer, Jiachen Huang, Berk A. Alpay, Larance Ronsard, Zhenfei Xie, Julianna Han, Monica Fernandez-Quintero, Quynh Anh Phan, Rebecca L. Ursin, Mya Vu, Kathrin H. Kirsch, Thavaleak Prum, Victoria C. Rosado, Thalia Bracamonte-Moreno, Vintus Okonkwo, Julia Bals, Caitlin McCarthy, Usha Nair, Masaru Kanekiyo, Andrew Ward, Aaron G. Schmidt, Facundo D. Batista, Daniel Lingwood

**Affiliations:** 1 The Ragon Institute of Mass General, The Massachusetts Institute of Technology and Harvard University, 400 Technology Square, Cambridge, MA 02139.; 2 Department of Integrative Structural and Computational Biology, The Scripps Research Institute, La Jolla, CA 92037.; 3 Systems, Synthetic, and Quantitative Biology Program, Harvard University, Cambridge, MA 02138.; 4 Department of Organismic and Evolutionary Biology, Harvard University, Cambridge, MA 02138.; 5 Department of General, Inorganic and Theoretical Chemistry, Center for Chemistry and Biomedicine, University of Innsbruck, Innrain 80-82/III, 6020, Innsbruck, Austria.; 6 Vaccine Research Center, National Institute of Allergy and Infectious Diseases, National Institutes of Health, 40 Convent Drive, Bethesda, MD 20892-3005.; 7 Department of Microbiology, Harvard Medical School, Boston, MA 02115, USA.; 8 Department of Biology, The Massachusetts Institute of Technology, Cambridge, MA 02139, USA

## Abstract

Broadly neutralizing antibodies (bnAbs) targeting the hemagglutinin (HA) stem of influenza A viruses (IAV) tend to be effective against either group 1 or group 2 viral diversity. In rarer cases, intergroup protective bnAbs can be generated by human antibody paratopes that accommodate the conserved glycan differences between the group 1 and group 2 stems. We applied germline-engaging nanoparticle immunogens to elicit a class of cross-group bnAbs from physiological precursor frequency within a humanized mouse model. Cross-group protection depended on the presence of the human bnAb precursors within the B cell repertoire, and the vaccine-expanded antibodies enriched for a N55T substitution in the CDRH2 loop, a hallmark of the bnAb class. Structurally, this single mutation introduced a flexible fulcrum to accommodate glycosylation differences and could alone enable cross-group protection. Thus broad IAV immunity can be expanded from the germline repertoire via minimal antigenic input and an exceptionally simple antibody development pathway.

## eTOC

Differences in glycosylation prevent antibodies from widely engaging the otherwise conserved hemagglutinin (HA) stem of influenza A viruses (IAV). Ray et al. deploy a humanized mouse to elicit a broadly neutralizing antibody (bnAb) response that results in group 1 and group 2 IAV protection via a single amino acid mutation that tilts the immunoglobulin to accommodate glycan diversity.

## Graphical Abstract

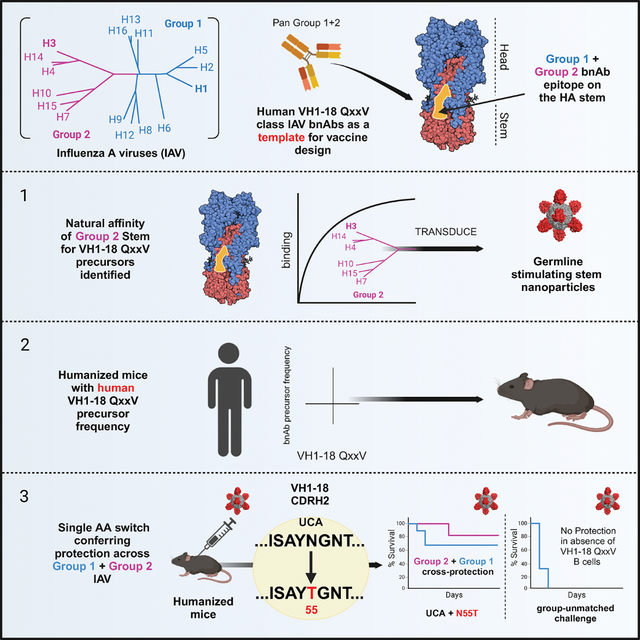

## Introduction

Influenza virus is a highly mutable pathogen that presents an ongoing diversity challenge to the immune system. A core issue is that hemagglutinin (HA), the major viral spike protein and seasonal vaccine immunogen, preferentially elicits antibodies against its hypervariable regions, limiting coverage and compromising pandemic preparedness^1–4^. Of the four influenza virus types (A, B, C, D), influenza A viruses (IAV) are responsible for a majority of infections in humans and are the source of influenza pandemics^5–7^. IAVs are phylogenetically divided into group 1 and group 2 viruses, and intragroup diversity is further subdivided into subtypes categorized by 18 distinct IAV HAs^8,9^. Broadly neutralizing antibodies (bnAbs) covering different levels of IAV diversity can be generated by targeting invariant features of HA, however these responses are immune-recessive/subdominant and will require rationally designed immune-focusing concepts to elicit higher titers^1,2,10–13^.

The majority of HA sequence diversity resides in its globular head domain, and ‘universal’ vaccine concepts currently under clinical evaluation include antibody-focusing to the relatively conserved stem or stalk domain of this protein^14–16^. These vaccines involve structure-based presentation of stem-only nanoparticles or sequential immunization with chimeric/head domain varying HAs to boost serum antibodies against conserved stem epitopes. Monoclonal human bnAbs can engage these sites to provide broad levels of coverage: heterosubtypic and the more exceptional cases of cross-group protection^2,17–25^. Within preclinical vaccine models, protective activity likely involves infection-blocking and Fc-mediated effector functions that are maximized when the bnAb sites are engaged^26–29^.

A feature of vaccine expanded stem responses in preclinical models is that coverage remains heterosubtypic and is not cross-group^28,30–33^. Differences between the glycosylation positions on the group 1 vs group 2 stem also impose steric constraints that hinder intergroup-reactivity to bnAb supersites^32,34,35^. There is no single stem immunogen that has been shown capable of eliciting intergroup IAV bnAbs. Rather, intergroup coverage has required co-formulation of group 1 + group 2 stem immunogens^36^.

In this study, we sought to elicit intergroup protective antibodies using a single vaccine immunogen by expanding germline BCR precursors that give rise to these bnAbs in humans. Functionally convergent human intergroup bnAbs are generated through public/shared antibody classes marked by usage of specific V_H_ genes, D_H_ genes and/or CDRH3 signatures^20,22,24,25^ and they have the potential to be triggered and expanded *in vivo* by different stem-focusing vaccine modalities^37,38^. We applied our one-step CRISPR/Cas9-induced homology-directed repair (HDR) platform^39,40^ to produce a humanized mouse vaccine model containing physiologically relevant recombination frequency of germline BCRs giving rise to intergroup bnAbs of the VH1–18 QxxV class^24,37^. We then immunized this mouse with recombinant nanoparticle displays of the group 2 HA stem^37^, which we found harbor natural binding affinity to VH1–18 QxxV bnAb precursors. The particles induced protection against group-unmatched IAV, and this was dependent on the bnAb precursors within our humanized mice. We found that after a single immunization, bnAb precursors were recruited to B cell germinal centers (GC) and underwent somatic hypermutation (SHM). Within 28 days post vaccination, potent and cross-protective bnAbs were elicited, and with only a fraction of mutations seen in the mature antibody from humans. Notably the immunogens enriched for N55T, a CDRH2 mutation present in all human VH1–18 QxxV bnAbs. We show that *this mutation alone* enables cross-group protection by providing a flexible fulcrum to pivot the antibody and accommodate the conserved glycosylation differences between the group 1 and 2 HA stems. Our findings thus reveal a simple molecular switch that can be triggered with minimal antigenic input to elicit exceptionally broad humoral immunity against IAV.

## Results

### VH1–18 QxxV bnAb precursors harbor natural affinity for HA stems from group 2 IAV

**Figure 1.**
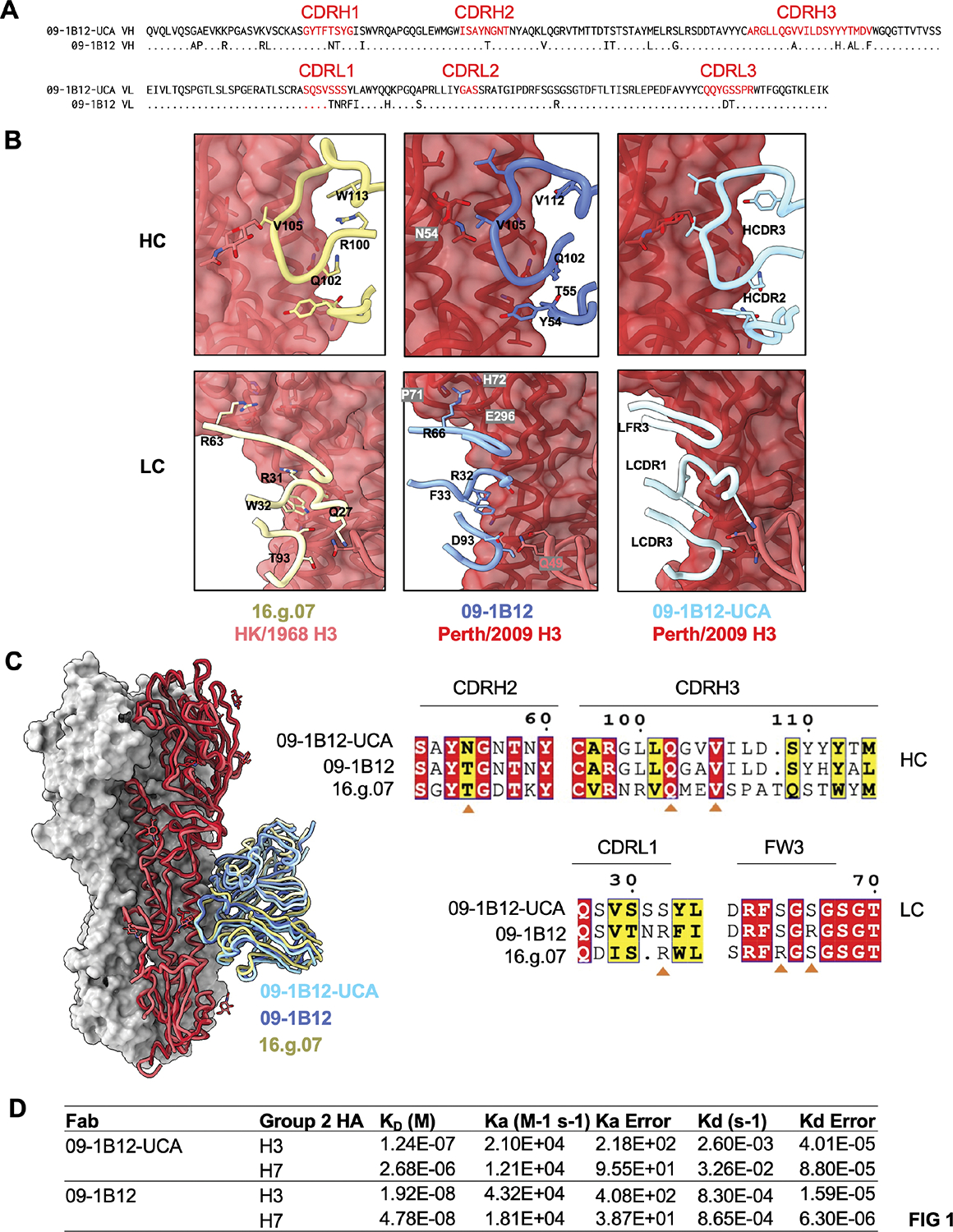
Germline VH1–18 QxxV bnAb shows natural affinity for HA from group 2 IAV. (A) Alignment of V_H_ and V_L_ amino acid sequences of 09–1B12-UCA vs mature 09–1B12. (B) CryoEM structure of 09–1B12-UCA and crystal structure of 09–1B12 in complex with H3 trimer bearing the H3ssF stem sequence (A/Perth/16/2009= red). Additional comparison is made with 16.g.07 (5KAN), another VH1–18 QxxV class bnAb^20^ (with H3 trimer A/Hong Kong/1/1968 = pink). (C) Overlay of the three antibodies along with conservation of antibody contacts sites in their HC and LC sequences (orange triangle). (D) Affinities of the germline versus mature forms of 09–1B12 for group 2 HA trimers (H3 from A/Perth/16/2009 and H7 from A/Shanghai/02/2013) [global fitting from four dilution curves for each antibody type (0.625 mM, 1.25 mM, 2.5 mM, and 5 mM), one experiment]. The HC and LC nucleotide sequences of 09–1B12-UCA was derived previously^37^. Natural gHgL affinities for H7 and H3 trimers are also seen for multiple VH1–18 QxxV bnAb class members (10^−6^-10^−8^ M) (Table S3). See also Figure S1 and Tables S1, S2 (in relation to B,C).

We began by examining the structural biology underscoring a prototypic pathway for development of VH1–18 QxxV class bnAbs: the elicitation of 09–1B12, a cross-group protective bnAb expanded in humans ^24,37^ (Figures 1A–C, S1, Tables S1, S2). Notably, the germline-inferred configuration of this antibody, 09–1B12-UCA, has reactivity to group 2 HA stems^37^ (see also Figure 1D) and we found that natural affinity for group 2 HA (H3 trimer from A/Perth/16/2009 and H7 trimer from A/Shanghai/02/2013) is also a generic feature of inferred VH1–18 QxxV bnAb precursors (10^−6^-10^−8^M) (Table S3). We leveraged this property to generate a 2.8 Å high resolution cryo-EM structure of 09–1B12-UCA in complex with the H3 trimer (A/Perth/16/2009) (Figure 1B,C). Comparison with the crystal structure of the mature 09–1B12, along with 16.g.07 (5KAN), another VH1–18 QxxV class bnAb^20^ indicates that germline affinity to the group 2 H3 stem is supplied by hallmark contacts of the VH1–18 QxxV class^20,24,38^: engagement via the “QxxV” positions in the CDRH3 along with V_H_-gene encoded contact by CDRH2 (Y54 and N/T55) (Figures 1B,C, S1). In addition, 16.g.07-like contacts in the antibody light chain CDRL1 (S/R at amino acid position 32) and FRW3 (S/R at amino acid positions 64, 66) were observed for both mature and UCA forms of 09–1B12 (Figure 1B,C, S1). The UCA also shares a 16.g.07-like CDRL1 (Q27) contact with the neighboring HA protomer (Q49), while in 09–1B12, the CDRL3 loop (D93 and T94) shows closer proximity to HA Q49 (Figure 1B,C). Collectively, these data point to the hallmark structural features of VH1–18 QxxV bnAbs as also responsible for the natural affinity between the bnAb precursor forms of this class and group 2 HA stems.

### Generating a humanized mouse model that contains a physiologically relevant frequency of VH1–18 QxxV bnAb precursors within the B cell repertoire

**Figure 2.**
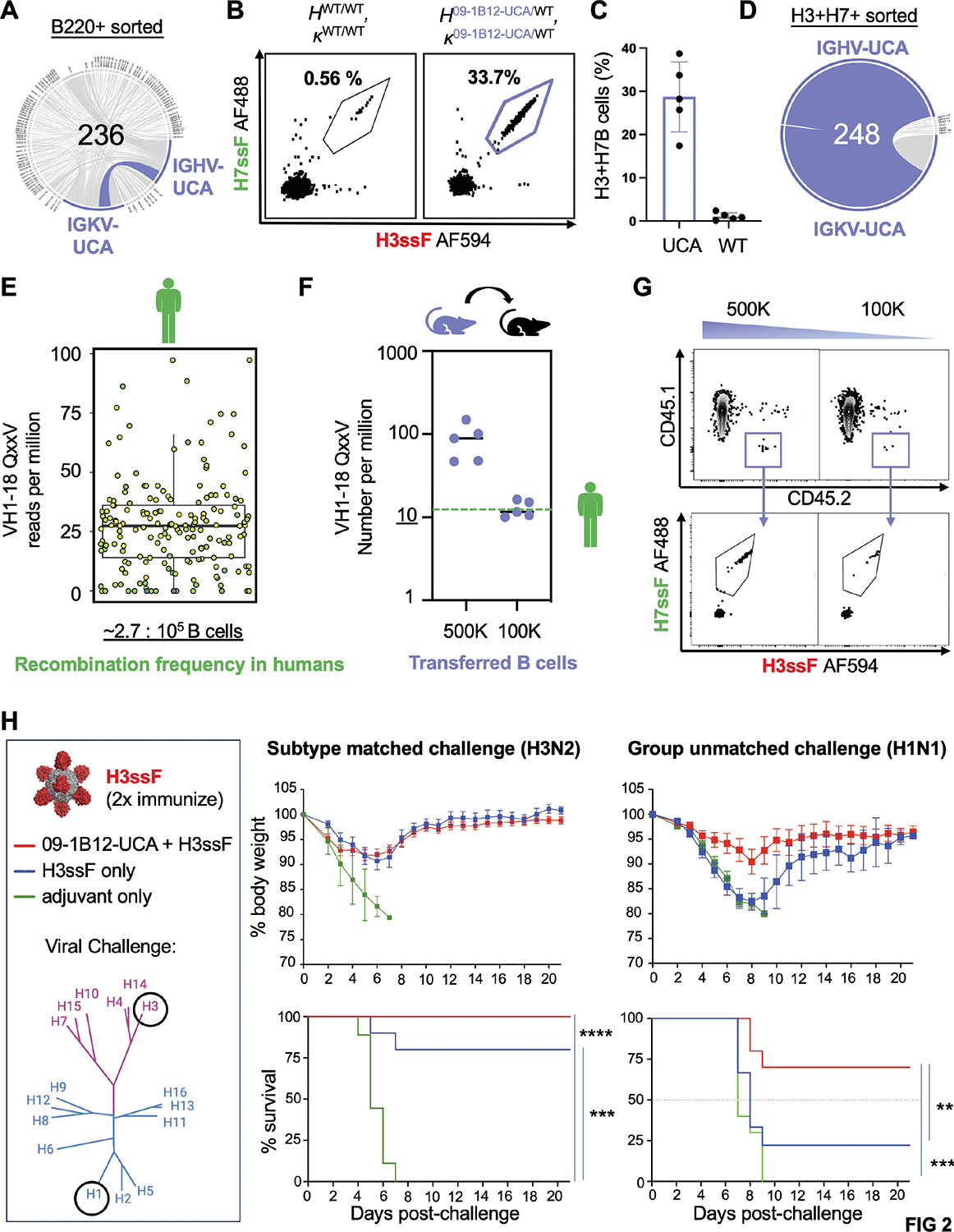
A group 2 HA stem immunogen elicits vaccine protection from group-unmatched influenza virus, but only when VH1–18 QxxV bnAb precursors are present at physiological frequency. (A) HC and LC sequencing of B220^+^ cells from CD45.2 09–1B12-UCA donor mice (*H*^09–1B12-UCA/WT^, *κ*^09–1B12-UCA/WT^) (one mouse, one experiment). Of these cells 11.02% were paired IGHV/IGKV for the VH1–18 QxxV 09–1B12-UCA sequence. (B) B220+ B cell cross-reactivity to H3ssF-AF594 and H7ssF-AF488 in WT C57Bl/6 versus 09–1B12-UCA donor mice. These cross-reactive B cells were also not specific for the ferritin scaffold as they did not bind H3ssF-KO or H7ssF-KO (central stem epitope KO = N-linked glycan at 45_HA2_). (C) Proportion of the H3^+^/H7^+^ cross-reactive B cells in WT C57Bl/6 versus 09–1B12-UCA donor mice (H3ssF^+^/H7ssF^+^/ H3ssF-KO^−^/H7ssF-KO^−^) (n=5, mean ± SD, one experiment). (D) Enrichment of the VH1–18 QxxV UCA in cross-reactive B cells was observed at 83.5% (one mouse, one experiment). (E) Box plot of frequencies of the VH1–18 QxxV bnAb precursors among IgM antibody repertoires of n=10 human subjects^41^. A box plot containing the biological and technical replicates is presented; a separate breakdown of these values in each individual human subject is presented in Figure S4A. From this data we obtain a median bnAb precursor frequency of 2.7 per 100,000 B cells. (F,G) To recapitulate this value in the B cell repertoires of CD45.1 mice, we transferred CD45.2 09–1B12-UCA B cells (*H*^09–1B12-UCA/WT^, *κ*^09–1B12-UCA/WT^) at increasing amounts and then measured the VH1–18 QxxV bnAb precursor frequency (H3ssF^+^/H7ssF^+^/ H3ssF-KO^−^/H7ssF-KO^−^) in the spleen of the recipient mice. Transfer of 100,000 09–1B12-UCA B cells reproducibly gave a value of ~1 in 100,000 B cells (n=5 recipient mice per transfer amount, one experiment), consequently this was the VH1–18 QxxV bnAb precursor frequency used in all subsequent immunization experiments. (H) 09–1B12-UCA B cells were transferred (or not transferred) to recipient mice that were 2x sequentially immunized with H3ssF (day 0 prime + day 42 boost) and then lethally challenged (day 56) with subtype matched H3N2 influenza virus [10^8^ TCID_50_/ml X-31] or group unmatched H1N1 influenza virus [10^4^ TCID_50_/ml mouse-adapted A/California/2009 (maA/Cal/09)^75,76^]. The genetic distance of these influenza virus subtypes is also shown by the accompanying phylogenetic tree of HA IAV diversity (group 2 = purple, group 1 = blue). Weight loss and survival were monitored over 21 days. For subtype-matched H3N2 challenge: ****P<0.0001 between 09–1B12-UCA cells + H3ssF versus Sigma adjuvant; ***P<0.001 between H3ssF alone vs Sigma adjuvant (n=10 mice per group, Mantel-Cox test of survivorship, one experiment). For group-unmatched H1N1: **P<0.025 between 09–1B12-UCA cells + H3ssF vs H3ssF alone; ***P<0.001 between 09–1B12-UCA cells + H3ssF vs Sigma adjuvant alone, where the n=2 surviving from H3ssF only group (absence of 09–1B12-UCA cells) was not statistically significant (P>0.05) (n=10 mice per group, Mantel-Cox test of survivorship, one experiment). See also Figure S2 and S3 (in relation to A–D) and Figure S4 (in relation to E).

Within pre-clinical vaccine models, generating cross-group protective IAV coverage has required co-formulation of group 1 + group 2 stem immunogens^36^. We developed a humanized mouse model to define whether individual stem immunogens could now elicit cross-group protective antibodies when VH1–18 QxxV class precursors were present, and at physiologically relevant frequency. In the first step, a 09–1B12-UCA KI mouse (*H*^09−1B12-UCA/WT^, *κ*^09−1B12-UCA/WT^) was generated using our CRISPR/Cas9-induced homologous directed recombination method ^39,40^ (Figures 2A–D, S2, S3). This mouse contained ~11% of paired HC + LC 09–1B12-UCA BCRs within the B220^+^ B cell repertoire (Figure 2A, S2A,B) and showed WT C57Bl/6-like B cell development in the bone marrow and periphery (Figure S3A–D). Importantly, we found that B220^+^ B cell cross-reactivity to H3ssF and H7ssF, two structure-guided group 2 stem-only ferritin nanoparticle immunogens derived from A/Perth/16/2009 and A/Shanghai/02/2013^37^, was markedly elevated in the *H*^09−1B12-UCA/WT^, *κ*^09−1B12-UCA/WT^ genotype relative to WT C57Bl/6 (Figure 2B,C). This cross-reactivity was specific to the central stem, as it did not include reactivity to epitope KO probes (H3ssF^+^/H7ssF^+^/H3ssF-KO^−^/H7ssF-KO^−^, where KO = N-linked glycan at 45_HA2_) (Figure S2A). H3ssF^+^/H7ssF^+^/H3ssF-KO^−^/H7ssF-KO^−^ B cells were also >80% enriched for the UCA sequence, demonstrating the capacity of the H3ssF and H7ssF immunogens to select for this germline BCR from a diverse B cell repertoire (Figure 2C,D, S2A,C).

To construct a humanized mouse model containing physiological bnAb precursor frequency, we measured recombination frequency of VH1–18 QxxV bnAb precursors within a publicly available IgM database of heavy-chain repertoire-sequenced human subjects ^41^ (Figure 2E, S4). In these subjects, VH1–18 QxxV bnAb precursors were found with a median recombination frequency of ~2.7 in 100 000 B cells (Figure 2E, S4), similar to the value reported for VH6–1 class influenza bnAb precursors ^42^. We then performed adoptive transfer of B cells from *H*^09−1B12-UCA/WT^, *κ*^09−1B12-UCA/WT^ to CD45.1 C57Bl/6 mice and measured the resultant H3ssF^+^/H7ssF^+^/H3ssF-KO^−^/H7ssF-KO^−^/CD45.2^+^/CD45.1^−^ B cells (= bnAb precursor) in the recipient animals (Figure 2F,G). There was a dose-response relationship between the number of B cells transferred and the VH1–18 QxxV bnAb precursor frequency established within the recipient spleen, akin to other humanized mouse systems deploying adoptive B cell transfer^39,40,43–45^. To match humans, we set the VH1–18 QxxV bnAb precursor frequency to 1 in 100 000 B cells in all downstream immunization / immune challenge experiments. Collectively, this established a humanized mouse model containing VH1–18 QxxV bnAb class precursors at a B cell repertoire frequency that matched VH1–18 QxxV recombination frequency seen in humans.

### Physiological B cell repertoire frequency of VH1–18 QxxV bnAb precursors enables vaccine-protection from group-unmatched IAV

H3ssF elicits heterosubtypic immunity within group 2 IAV, but this does not extend to group 1 IAV^37^; coformulation of group 1 and group 2 immunogens is needed for cross group protection^36^. To initially define whether the presence of VH1–18 QxxV bnAb precursors (at human-like recombination frequency, see also Figure 2E–F) could enable cross group protection, we sequentially (2x) immunized animals containing (or not containing) 09–1B12-UCA B cells (at human precursor frequency) with H3ssF (+ Sigma adjuvant) and then lethally challenged with subtype-matched H3N2 or group-unmatched H1N1 influenza viruses (Figure 2H). We found that H3ssF protected against subtype-matched H3N2 virus in the absence of UCA cells, but required their presence for protection against the group-unmatched H1N1 virus. Hence, the inclusion of VH1–18 QxxV bnAb precursors into a diverse host B cell repertoire at human-like recombination frequency enabled the elicitation of cross-group protective immunity, all using a single recombinant vaccine immunogen.

### Individual stem nanoparticles selectively expand and mature the bnAb precursors within B cell germinal centers in the humanized mice

**Figure 3.**
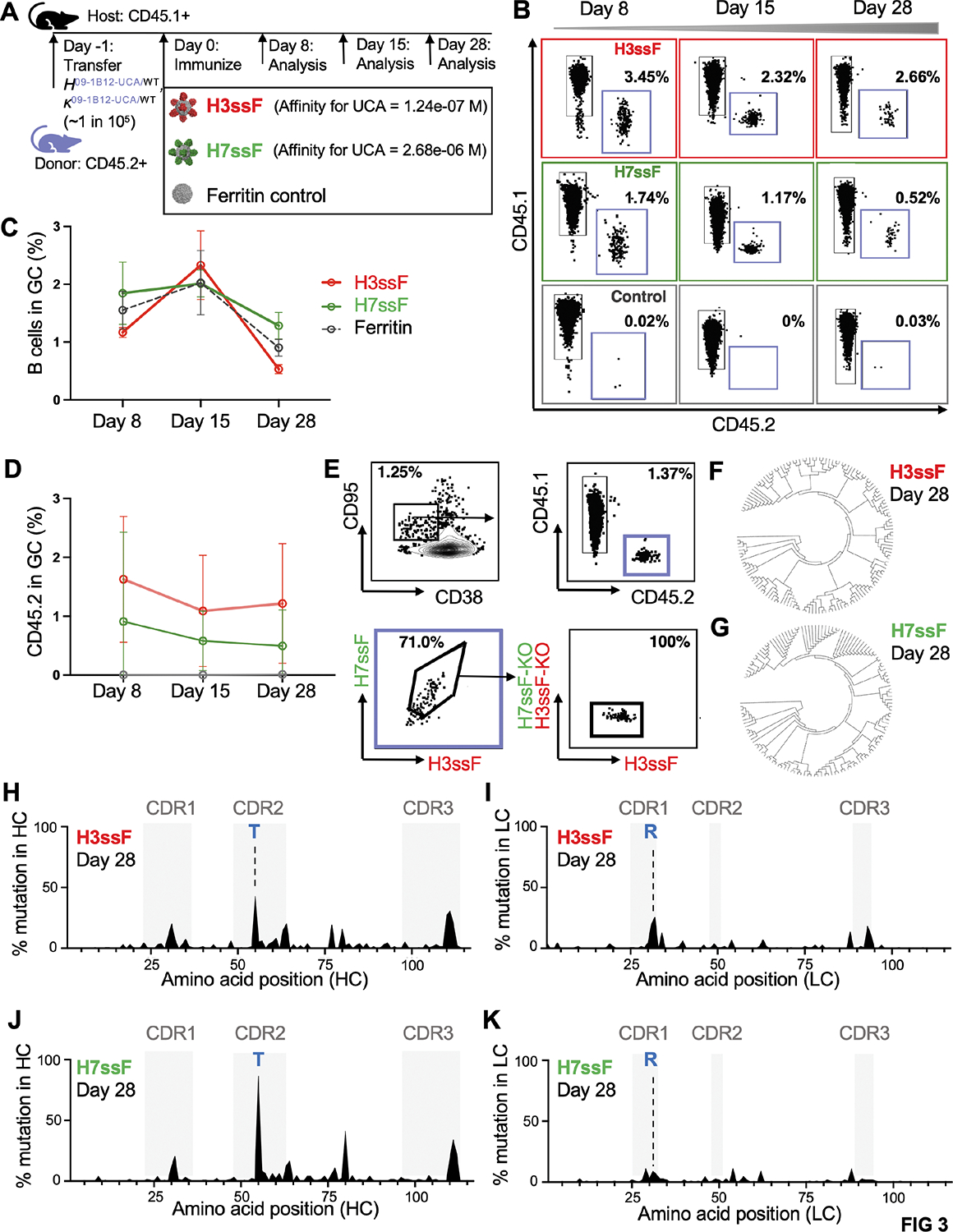
Stem nanoparticles selectively expand VH1–18 QxxV bnAb precursors from physiological frequency in the antibody repertoire and induce diversification through somatic hypermutation in GCs. (A) Schematic of adoptive transfer performed at precursor frequencies of ~1 per 10^5^ 09–1B12-UCA B cells into WT mice at day −1 and subsequent single immunization of higher affinity H3ssF or lower affinity H7ssF. Naked ferritin particles were also given as a control. All vaccines were adjuvanted by the Sigma Adjuvant System. Spleens were sampled at the time points indicated. (B) Representative flow plots of CD45.2 B cells being recruited to GCs at days 8, 15, and 28 post-vaccination. (C) The percentage of GC B cells in the CD45.1 host was quantified at each time point (n=5 mice per immunogen, mean ± SD, one experiment). (D) The percentage of CD45.2 B cells within the host GCs each time point (n=5 mice per immunogen, mean ± SD, one experiment). (E) The GC CD45.2 B cells were also marked by epitope specificity to the central stem site [H3ssF^+^/H7ssF^+^/H3ssF-KO^−^/H7ssF-KO^−^ (central stem epitope KO =N-linked glycan at 45_HA2_)] and single GC CD45.2 B cells in this gate were sorted by FACS and subjected to BCR sequencing. Results presented in B-E were recapitulated if H7 and H3 trimers were used (instead of nanoparticles) as the antigen B cell probes (Figure S5C–G). (F, G) HC nucleotide diversification of H3ssF^+^/H7ssF^+^/H3ssF-KO^−^/H7ssF-KO^−^ B cell clones at 28 days after immunization with H3ssF or H7ssF. (H) Mutation frequency in the HC amino acid sequence at 28 days post immunization with H3ssF. (I) Mutation frequency in the paired LC amino sequence at 28 days post immunization with H3ssF. (J) Mutation frequency in the HC amino sequence at 28 days post immunization with H7ssF. (K) Mutation frequency in the paired LC amino sequence at 28 days post immunization with H7ssF. (H-K) Blue letters mark enrichment of amino acid mutations also present in mature 09–1B12 and 16.g.07. Data from (F-K) is from 125 GC BCRs for H3ssF (n=3 mice), 111 GC BCRs for H7ssF (n=3 mice); one experiment. See also Figure S5 (in relation to A–E) and Table S4 (in relation to F–K).

To investigate the B cell pathways underscoring cross-group protection, we tracked CD45.2 VH1–18 QxxV UCA lineage expansion after a single immunization. In these experiments, we deployed H3ssF and H7ssF^37^ as higher and lower affinity germline stimulating immunogens (Figure 3A); monomeric UCA BCR affinities for the H3 and H7 stems from these antigens is separated by a logfold difference (1.24e-07 vs 2.68e-06 M) (see also Figure 1D, Table S3). H3ssF and H7ssF (+Sigma adjuvant) triggered comparable recruitment of CD45.2 B cells to germinal centers (GCs) within the CD45.1 hosts after one immunization step (Figure 3B–D, Figure S5A,B). Naked ferritin particles were also injected with Sigma Adjuvant (Figure 3A) and while this elevated GC reactions in the CD45.1 host (Figure 3B,C), it was not accompanied by recruitment of CD45.2 UCA B cells (Figure 3D). Thus, expansion of the CD45.2 UCA B cells was dependent on the stem-antigens. We further confirmed that GC-recruited CD45.2 B cells were targeting the central stem epitope (H3ssF^+^/H7ssF^+^/H3ssF-KO^−^/H7ssF-KO^−^, epitope KO = insertion of N-linked glycan at 45_HA2_) (Figure 3E, S5A,B). If we exchanged our nanoparticle flow cytometry probes for H7 and H3 trimers, we recapitulated our key findings: expansion of H3^+^/H7^+^ cross-reactive CD45.2 B cells into GCs at 8, 15 and 28 days after immunizing with H3ssF or H7ssF, and failure to trigger this response if ferritin alone is deployed as the immunogen (Figure S5C–G).

Single cell BCR sequencing of the CD45.2^+^/CD45.1^−^/H3ssF^+^/H7ssF^+^/H3ssF-KO^−^/ H7ssF-KO^−^ GC B cells revealed diversification through SHM in response to both H3ssF and H7ssF (Figure 3F–K; Table S4). SHM was concentrated in the CDR1–3 regions of the HC and LC (Figure 3H–K), and mutations also accumulated in FW3 of the HC and LC (Figure 3H–K). Notably, the mutations N55T (CDRH2) and S32R (CDRL1) enriched in the vaccine-expanded BCRs (Figure 3H–K) are also present in the mature 09–1B12 and 16.g.07 (Figure 1B,C). In the mature antibodies, contact to the group 1 stem is greatly strengthened by S32R in CDRL1 (Figure 1). The importance of N55T, a hallmark of the VH1–18 QxxV bnAb class in humans^24^, is detailed in the following sections. Collectively, these data indicate that our immunogens: (A) selectively trigger and expand the VH1–18 QxxV bnAb lineage from a diverse B cell repertoire bearing bnAb precursors at human-like recombination frequency; and (B) guide SHM and affinity selection to enrich for some hallmark mutations seen for this bnAb class in humans.

### The stem nanoparticles elicit cross-group protective bnAbs with minimal SHM after a single immunization.

**Figure 4.**
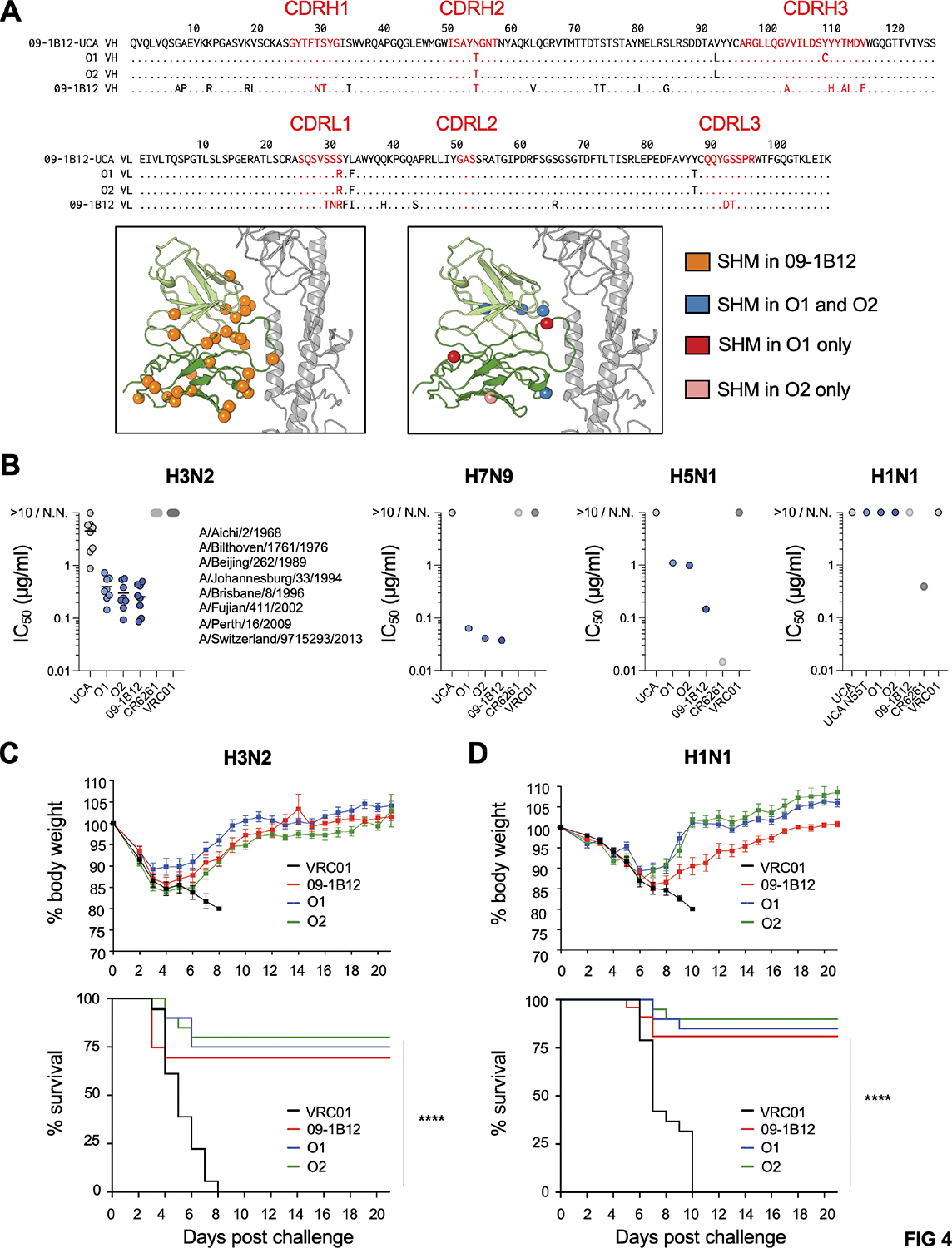
Vaccine expansion of cross-group protective IAV bnAbs that use minimal SHM. (A) O1 and O2 were example mAbs expanded after a single immunization with H3ssF or H7ssF, respectively. O1 and O2 sequences contain some of the mutations present in mature 09–1B12, but have far lower SHM, as noted in the amino acid alignments and antibody structure. (B) Neutralization activities of 09–1B12-UCA, O1, O2 and 09–1B12 across: H3N2 (eight diverse viral strains covering >50 years of diversification: A/Aichi/2/1968, A/Bilthoven/1761/1976 A/Beijing/353/1989, A/Johannesburg/33/1994, A/Brisbane/8/1996, A/Fujian/411/2002, A/Perth/16/2009, A/Switzerland/9715293/2013); H7N9 (A/Shanghai/02/2013); H5N1 (A/Vietnam/1203/2004); and H1N1 (A/California/07/2009). Cross-group protecting VH1–18 QxxV bnAbs neutralize group 2 viruses but show more limited neutralizing activity against group 1 IAV^24,46^. VRC01 served as an isotype control, 09–1B12 was a positive control for group 2 IAV neutralization and CR6261 was a positive control for group 1 IAV neutralization. N.N. = non-neutralizing (each antibody was run in triplicate, one experiment). (C) Weight loss and survival from lethal H3N2 virus challenge (10^8^ TCID_50_/ml X-31) following passive transfer of 5 mg/kg O1, O2, 09–1B12 or VRC01 as isotype control [n=20 mice per group, ****P<0.001 (Mantel-Cox test of survivorship), one experiment]. (D) Weight loss and survival from lethal H1N1 challenge [10^4^ TCID_50_/ml maA/Cal/09^75,76^] following passive transfer of 5 mg/kg O1, O2, 09–1B12 or VRC01 as isotype control [n=20 mice per group, ****P<0.0001 (Mantel-Cox test of survivorship), one experiment]. See also Table S4 (in relation to A).

We expressed two example mAbs (O1 and O2) that were expanded by H3ssF or H7ssF, respectively (Figure 4A, Table S4). These antibodies harbored some of the amino acid mutations present in mature 09–1B12, however they also showed far lower SHM (Figure 4A). Despite this, these vaccine elicited antibodies showed 09–1B12-equivalent activities: (1) comparable neutralization activity across group 2 IAV, along with more limited neutralization of group 1 IAV, as expected for the VH1–18 QxxV bnAb class^24,46^ (Figure 4B); (2) comparable protection against H3N2 viral challenge following passive transfer to C57Bl/6 WT mice (Figure 4C); and (3) comparable protection against H1N1 viral challenge following passive transfer to C57Bl/6 WT mice (Figure 4D). These results indicate that cross-group protective antibodies were elicited after a single immunization, and achieved fully functional somatic activity with minimal SHM.

### Enrichment of N55T within the CDRH2 of germinal center BCRs and serum antibodies reveals a simple molecular switch conferring group 1 + group 2 IAV protection

**Figure 5.**
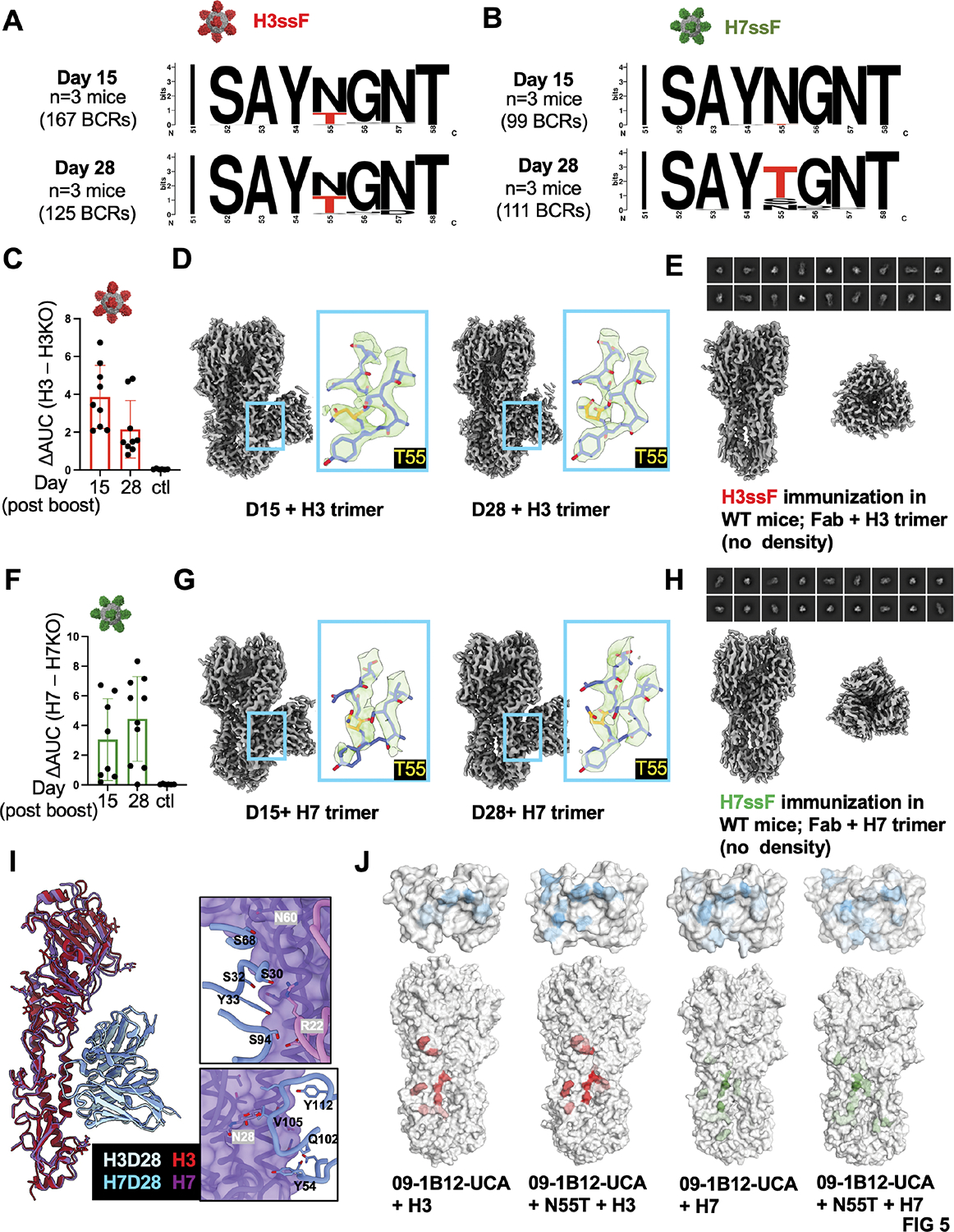
Stem focused VH1–18 QxxV responses enrich for the public mutation N55T within germinal centers and serum antibodies elicited by stem nanoparticles. (A, B) Logo plots of the CDRH2 domain of the VH1–18 QxxV class precursors enriched in the GCs at 15 and 28 days post-immunizations with H3ssF or H7ssF (see also Figure 3F–K; 167 GC BCRs for H3ssF at Day 15 (n=3 mice), 125 GC BCRs for H3ssF at Day 28 (n=3 mice), 99 GC BCRs for H7ssF at Day 15, 111 GC BCRs for H7ssF at Day 28 (n=3 mice); one experiment). (C-I) CryoEMPEM was performed on VH1–18 QxxV antibodies elicited in the serum after sequential immunization (day 0 prime + day 42 boost) with either H3ssF or H7ssF. In all cases immune sera were evaluated at 15 and 28 days post-boost. (C) H3ssF elicited IgG showing differential reactivity to H3 ± epitope KO (central stem epitope KO= N-linked glycan at 45_HA2_); adjuvant only is the control (n=9 at Day 15 and n=9 mice at Day 28; n=6 control mice received adjuvant only; one experiment). (D) CryoEMPEM of H3ssF immune sera at 15 and 28 days post-boost, with antibodies in complex with H3 trimer (A/Perth/16/2009) [immune sera pooled from all mice at Day 15 (one experiment); immune sera pooled from all mice at Day 28 (one experiment)]. The 09–1B12-UCA Fab was docked into the maps and the HC N55 residues are boxed in red. (E) No density of 09–1B12-like Fab in complex with H3 trimer was found in the immune sera from WT C57Bl/6 mice (lacking VH1–18 QxxV bnAb precursors) subjected to the same H3ssF immunization regimen (28 days post-boost is shown) (immune sera pooled from n= 10 mice, one experiment). (F) H7ssF-elicited IgG showing differential reactivity to H7 ± epitope KO (central stem epitope KO= N-linked glycan at 45_HA2_); adjuvant only is the control (n=8 mice at Day 15 and n=9 mice at Day 28; n=6 control mice received adjuvant only; one experiment). (G) CryoEMPEM of H7ssF immune sera at 15 and 28 days post-boost, with antibodies in complex with H7 trimer (A/Shanghai/02/2013) [immune sera pooled from all mice at Day 15 (one experiment); immune sera pooled from all mice at Day 28 (one experiment)]. The 09–1B12-UCA Fab was docked into the maps and the HC T55 residues are yellow. (H) No density of 09–1B12-like Fab in complex with H7 trimer was found in the immune sera from WT C57Bl/6 mice (lacking VH1–18 QxxV bnAb precursors) subjected to the same H7ssF immunization regimen (28 days post-boost is shown) (immune sera pooled from n= 10 mice, one experiment). (I) Overlapping cryo-EMPEM structure of H7ssF and H3ssF immune sera at 28 days post-boost [09–1B12-UCA Fab docked in; with LC contacts (upper panel) and HC contacts (lower panel) shown]. (J) MD simulations of 09–1B12-UCA in contact with H3 (A/Perth/16/2009) or H7 (A/Shanghai/02/2013) trimers ± N55T. The color gradient on the surface indicates the interaction time for hydrogen bonds and charged interactions (darker colors means longer interaction time). See also Table S4 (in relation to A,B) and Figure S1, Table S1 (in relation to D,G,I).

The H3^+^/H7^+^ cross-reactive 09–1B12-UCA BCRs expanded within GCs were enriched for the mutation N55T within the CDRH2 loop (Figure 3H,J, Table S4). Skewed use of this mutation was seen at 28 days post-immunization with H3ssF or H7ssF, but it also enriched earlier at 15 days when the higher affinity H3ssF was used as the immunogen (Figure 5A,B, Table S4). This enrichment was also observed in the serum antibodies elicited following sequential immunization with either H3ssF or H7ssF (day 0 prime + day 42 boost), as evaluated by cryoEMPEM ^47,48^ at 15 and 28 days post-boost (Figure 5C–H, S1, Table S1). At these timepoints, the serum antibodies had specificity to the central stem epitope, as judged by differential reactivity to H3ssF/H7ssF ± stem epitope KO (N-linked glycan at 45_HA2_) (Figure 5C,F). For cryoEMPEM, Fabs purified from the immune sera were complexed with HA trimers matched to H3ssF or H7ssF (A/Perth/16/2009 and A/Shanghai/02/2013, respectively), confirming antibody targeting to the central stem (Figure 5D,G, I). The significant differences of the local map resolutions at CDRH2 loop as well as the volumes around residue 55 between H3 and H7 complexes suggest H7 preferably binds to Fabs that harboring T55 but not N55, which is consistent with the binding affinity data (KDs of H7 binding to 09–1B12-UCA vs 09–1B12-UCA + N55T, Table S3). Notably, when the same H3ssF or H7ssF immunization regimens were applied to WT C57Bl/6 mice (not bearing 09–1B12-UCA cells), we failed to resolve Fab density for the full-length H3 or H7 trimer, further underscoring importance of B cell repertoire in eliciting antibodies against the central stem epitope (Figure 5E,H). When the bnAb precursors are in place, our results highlight N55T as a vaccine-selected mutation, both in the GC and serum antibody response.

**Figure 6.**
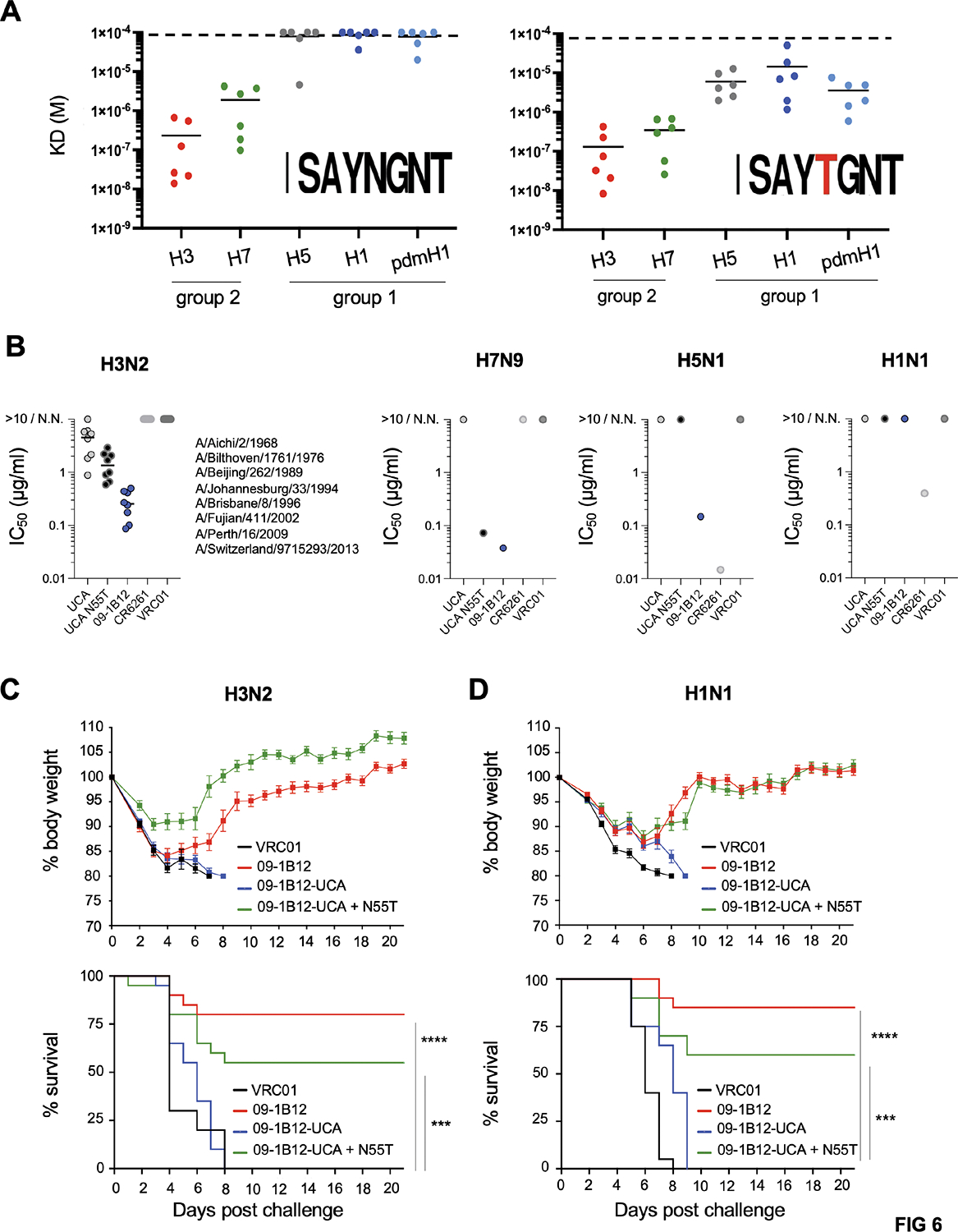
N55T alone enables cross-group recognition and protection. (A) Binding affinities for UCA-inferred Fabs from six VH1–18 QxxV bnAb class members ± N55T [09–1B12, 05–2A09, 05–2D04, 27–1D08, 21–1A01, 06–1F04] to group 1 and group 2 IAV HAs (H3 = A/Perth/16/2009; H7 = A/Shanghai/02/2013; H5 = A/Indonesia/05/2005; H1 = A/Michigan/45/2015; pdmH1 = A/California/07/2009) as measured by biolayer interferometry [global fitting from four dilution curves for each antibody type (0.625 mM, 1.25 mM, 2.5 mM, and 5 mM), one experiment]. Values above 100 uM are undetectable with our instrument^77^. (B) Neutralization activities of 09–1B12-UCA, 09–1B12-UCA + N55T, and 09–1B12 across: H3N2 (eight diverse viral strains covering >50 years of diversification: A/Aichi/2/1968, A/Bilthoven/1761/1976 A/Beijing/353/1989, A/Johannesburg/33/1994, A/Brisbane/8/1996, A/Fujian/411/2002, A/Perth/16/2009, A/Switzerland/9715293/2013); H7N9 (A/Shanghai/02/2013); H5N1 (A/Vietnam/1203/2004); and H1N1 (A/California/07/2009). Cross-group protecting VH1–18 QxxV bnAbs neutralize group 2 viruses but show more limited neutralizing activity against group 1 IAV^24,46^. N.N. = non-neutralizing. VRC01 served as an isotype control, 09–1B12 was a positive control for group 2 IAV neutralization and CR6261 was a positive control for group 1 IAV neutralization. The neutralization values obtained were generated within the same experiment shown in Figure 4B and therefore contain the same positive and negative control values (each antibody was run in triplicate, one experiment). (C) Weight loss and survival from lethal H3N2 virus challenge (10^8^ TCID_50_/ml X-31) following passive transfer of 5 mg/kg 09–1B12-UCA, 09–1B12-UCA + N55T, 09–1B12, or VRC01 as the isotype control [n=20 mice per group, ****P<0.0001, ***P<0.001 (Mantel-Cox test of survivorship)]. (D) Weight loss and survival from lethal H1N1 virus challenge [10^4^ TCID_50_/ml maA/Cal/09^75,76^] following passive transfer of 5 mg/kg 09–1B12-UCA, 09–1B12-UCA + N55T, 09–1B12 or VRC01 as isotype control [n=20 mice per group, ****P<0.0001, ***P<0.001 (Mantel-Cox test of survivorship)]. See also Table S3 (in relation to A) and Figure S6 (in relation to A–D).

Given that N55T is a marker of the VH1–18 QxxV bnAb class in humans^24^, we initially assessed the consequences of this mutation by performing molecular dynamics (MD) simulations of the 09–1B12-UCA in complex with H3 or H7 trimers (Figure 5J). In both scenarios T55 increased the interaction time and interaction areas of the QxxV motif, and also increased LC interactions with HA (Figure 5J). Accordingly, we biochemically evaluated the contribution of N55T to recognition of both group 2 and group 1 HAs for UCAs inferred from six different human VH1–18 QxxV class bnAbs (reversion of sHsL to gHgL) (Figure 6A). We found that for all antibodies, N55T strengthened germline-encoded antibody affinity to group 2 HA and also enabled binding to group 1 HAs, which was otherwise undetectable with the UCA (Figure 6A, Table S3). Alone, N55T also provided broad neutralizing activity against group 2 IAV (Figure 6B), a hallmark activity of mature VH1–18 QxxV bnAbs^24,46^. To define whether this single mutation alone enabled broad cross-group protection, we performed passive antibody transfer of 09–1B12-UCA mAb ± N55T into C57Bl/6 WT mice and then challenged with lethal doses of H3N2 or H1N1 viruses (Figure 6C,D). We observed a similar result after challenging from both viruses: the UCA form of the antibody failed to protect, however addition of N55T provided cross-group protection for >50% of recipient animals, approaching the activity of the mature 09–1B12 (Figure 6C,D). Hence, this single vaccine-selected mutation could alone enable cross-group protection from IAV.

### N55T provides a ‘fulcrum release’ to accommodate conserved group 1 N-glycans, enabling dual recognition of group 1 and group 2 stems.

**Figure 7.**
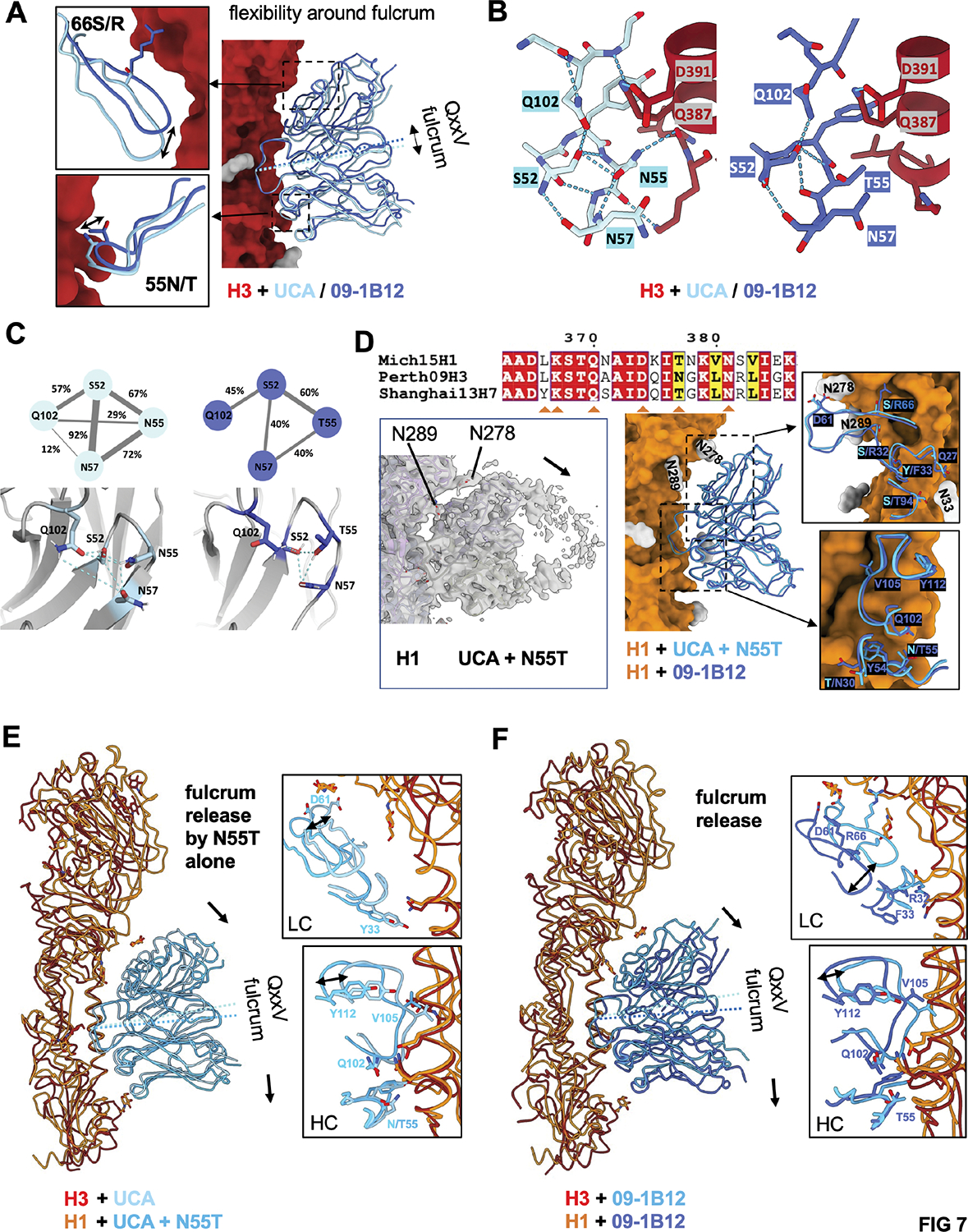
N55T provides a ‘fulcrum release’ to enable recognition of group 1 and group 2 HA stems. (A) Overlay of the 09–1B12-UCA and mature 09–1B12 in complex with H3 (A/Perth/16/2009; see also Figures 1B,C, S1, Tables S1, S2) illustrates the angular change where the HC and LC pivot around the QxxV fulcrum (=flexibility around the fulcrum). (B) N55 forms more hydrogen bonds with S52, N57, and Q102 in the CDRH2 and CDRH3 loops (visualized using ChimeraX). By contrast, T55 shows substantially fewer interactions to better accommodate changes in the binding angles. (C) MD simulations on the Fabs alone (without HA) show the percentages of interactions between paired residues (S52, N/T55, N57, and Q102) during simulations. (D) The cryoEM map of 09–1B12-UCA + N55T and overlayed structures of 09–1B12-UCA + N55T/09–1B12 in complex with H1 (A/Michigan/45/2015) (3.5 and 3.3 Å, respectively). A rotational tilt is imposed by group 1 IAV N-glycans at positions N289, N278, and N33 from the neighboring protomer interacting with the antibody LC. (E) N55T alone enables this tilting around the QxxV fulcrum (which we term fulcrum release) as visualized by overlay of H3 + 09–1B12-UCA co-complex with H1 + 09–1B12-UCA-N55T co-complex. (F) Fulcrum release as seen in the mature antibody as visualized by overlay of H3 + 09–1B12 co-complex with H1 + 09–1B12 co-complex. Arrows indicate the flexible antibody tilting enabled by fulcrum release to accommodate the conserved group 1 IAV glycan positions. See also Figure S1, Tables S1, S2.

By overlaying the complexes of H3 + UCA or 09–1B12, a shift in Fab binding angles was observed, driven by the movements of HC N55T and LC S66R mutations (Figure 7A, S1). Notably the CDRH3 loop (QxxV) is at the fulcrum of this rotation (Figure 7A). This flexibility around the QxxV fulcrum is underscored by our finding that N55 forms more hydrogen bonds with the other residues within CDRH2/3 loops (S52, N57, and Q102), while T55 showed fewer interactions with those residues (Figure 7B,C). Collectively, this indicates that the CDRH2 loop with T55 is more flexible to accommodate the changes in Fab binding angles. To determine the structural contribution of N55T for group 1 HA, we resolved a co-complex of H1 trimer (A/Michigan/45/2015) with 09–1B12-UCA + N55T (3.5 Å) and mature 09–1B12 (3.3 Å) (Figure 7D, S1). Notably, group 2 bnAbs can be prevented from recognizing the group 1 stem by clashing with conserved HA1 N-linked glycans^32,34,35^. We find that N55T driven flexibility enables dual recognition of group 1 HA through a mechanism we term ‘fulcrum release’. Here the group 1 IAV N-glycans at positions N289, N278, and N33 from the neighboring protomer interact with the antibody LC, tilting the antibody contact angle backward (Figure 7D). Fulcrum release enables UCA + N55T (Figure 7E) and 09–1B12 (Figure 7F) to accommodate this tilt and approach the central stem epitope and to avoid clashing with the group 1 N-glycans. Hence, we provide the structural basis for a single amino acid mutation in the germline encoded CDRH2 loop that enables VH1–18 QxxV antibodies to engage group 1 and group 2 HA stems.

### N55T is a generic feature that enables cross-group protection by VH1–18 QxxV bnAb class members.

Given that N55T is present in all VH1–18 QxxV bnAbs, we evaluated its contribution to cross-group protection by two other members of this bnAb class: 21–1A01; and 05–2A09^24^ (Figure S6). We passively transferred the mature and inferred UCA forms of these antibodies (± N55T) into C57Bl/6 WT mice and then challenged with H3N2 or H1N1 viruses (Figures S6A,B). Akin to our previous findings with 09–1B12 ± N55T (Figure 6C,D), the UCA forms were comparable to the isotype control, and failed to enable statistically significant protection. However, addition of N55T provided significant cross-group protection for >50% of the mice, approaching the activity of mature forms (Figures S6A,B). Hence, we conclude that N55T, identified by selection within the antibody responses of our humanized mouse system, provides a simple and general molecular ‘switch’ that enables cross-group protection by VH1–18 QxxV bnAbs.

## Discussion

Enhancing coverage of influenza virus diversity traditionally involves inclusion of more or different HA antigens in the seasonal vaccine^49,50^. This principle of additivity has been greatly extended by the recent development of an mRNA vaccine that encodes for HA representatives from all influenza A and B virus categories that elicits broad protection in mice^51^. By contrast, a central challenge for antibody-focusing concepts is to elicit broad coverage through a minimal set of rationally designed antigens^1,2^. Indeed, the compression of multiple protein functionalities within a single molecule is an important theme in the design of therapeutics and scalable biologics^52,53^. Our results highlight a simple molecular ‘switch’ that can be triggered by a single (and simple) recombinant HA immunogen to deliver exceptionally broad coverage across IAV.

In the absence of human bnAb precursors, HA nanoparticle immunogens (including H3ssF and H7ssF) can elicit heterosubtypic immunity against IAV subtypes, however these responses are not cross-group protective^28,30–32,37^. Under these conditions, elicitation of cross-group IAV antibodies relies on addition: co-formulation of group 1 + group 2 stem immunogens^36^. By contrast, our results demonstrate that vaccine-elicitation of cross-group protective immunity by a single stem immunogen can be enabled by specific germline-encoded features within the human antibody repertoire. We previously reported a human-repertoire prerequisite for vaccine-expanding group 1 bnAb classes within mice^28,54,55^ and there are analogous repertoire-requirements for expanding these bnAbs within non-human primates^33^. A low level of H3^+^/H7^+^cross-reactive B cells were initially present in the C57Bl/6 repertoire, however these cells likely lacked sufficient antigen affinity and/or frequencies to enable either vaccine protection against group-unmatched IAV or elicitation of high serum titers of central stem bnAbs that could be detected by cryoEMPEM. While there may be limits on our capacity to detect expansion of these host lineages, our results identify a decisive repertoire effect when VH1–18 QxxV bnAb precursors are present at physiological frequency, namely vaccine elicitation of cross-group protecting IAV bnAbs.

Both precursor frequency and BCR affinity for cognate antigen modulate recruitment to B cell GCs after immunization^43,45,56^. In our system, precursors could be expanded into cross-group protective bnAbs over a logfold range of germline affinities for the group 2 stem (10^−6^ - 10^−7^ M). Vaccine expansion of protective bnAbs across this affinity range was likely supported by fundamental avidity factors, such as stem nanoparticle valency and cell surface arrayed BCR^57–64^, and ultimately by the exceptionally low SHM requirement that we find is needed for full protective activity by VH1–18 QxxV class bnAbs. This minimal SHM greatly contrasts the complex affinity maturation pathways seen for human bnAbs against other hypervariable pathogens such as HIV^65,66^ and is exemplified by the N55T molecular switch enabling protection against group 1 and 2 IAV. The correlates of protection we observe are also consistent with VH1–18 QxxV bnAb class: broad neutralizing activity against group 2 viruses with non-neutralizing protection from group 1 IAV^24^.

The N55T substitution is not catalyzed by conventional mutation hotspots^67,68^, but it is nevertheless a marker of the human VH1–18 QxxV bnAb class^20,24^. Enrichment of N55T in the GCs expanded within our humanized mouse system pointed to a previously unrecognized functional importance for this signature that we then verified in other VH1–18 QxxV class bnAbs from humans. Our data indicates that this public amino acid substitution provides a ‘fulcrum release’ action that pivots the antibody to accommodate a conserved group 1 N-glycan and extend ‘hardcoded’ germline recognition of group 2-only to group 1 + 2 stems. Achieving this clash relief through a quite minor shift in the organization of the antibody paratope further underscores the low threshold for expanding this bnAb class.

It is uncertain how pre-existing immunity to HA antigens from prior infection and/or vaccination will modulate this simple pathway for bnAb development. Obligate recall of strain specific memory B cells through ‘primary addiction’ or related feedback effects has the potential to hamper expansion of the pathway^69–72^. However, the structurally similar group 1 stem nanoparticle, H1ssF, does successfully expand germline-encoded group 1 IAV bnAbs in humans^14,15^, suggesting that absence of an ‘immunodistractive’ HA head domain may be important for avoiding off-target memory recall when pre-existing immunity is present.

Collectively, our studies demonstrate proof-of-concept for vaccine expansion of unusually broad, cross-group protecting IAV bnAbs using a single recombinant immunogen. This is underscored by a simple molecular signature / switch that can be triggered from the human germline antibody repertoire to enable coverage through minimal immunological complexity.

### Limitations of study

There are additional contacts that support cross-group-protection by VH1–18 QxxV class bnAbs^20,24^ and we have not assigned their hierarchy in relation to fulcrum release. There are also orthogonal/additional, non-VH1–18 QxxV bnAb classes in humans that enable cross-group protection^20,22,24,25,38^ and it is not yet clear if single immunogens will be capable of collectively expanding these pathways and if these bnAbs are also enabled by minimal SHM. We have also used germline inferred BCRs as opposed to *bona fide* bnAb precursors, although we would note that rationally designed germline stimulating immunogens originally based on inferred UCA have thus far all succeeded in selectively expanding their ‘authentic’ counterparts in human clinical trials^14,15,31,73,74^. Lastly, and as mentioned earlier, have not accounted for prior exposure to influenza virus or seasonal vaccines where different individual immune-histories may skew ‘intended’ humoral immunity through ‘unintended’ memory recall^69–72^.

## STAR METHODS

### LEAD CONTACT AND MATERIALS AVAILABILITY

#### Lead Contact

Further information and requests for reagents should be directed to and will be fulfilled by the Lead Contact, Daniel Lingwood (dlingwood@mgh.harvard.edu).

#### Materials Availability

There are no restrictions on the availability on the materials used in this study.

#### Data and Code Availability

- 3D maps and models from the EM analysis have been deposited to the Protein Data Bank (http://www.rcsb.org/) and Electron Microscopy Databank (http://www.emdatabank.org/), respectively, and are publicly available from the date of publication. Accession numbers are listed in the Key Resources Table. Paired HC and LC sequences from the GCs expanded by H3ssF and H7ssF are deposited in Genbank. Accession numbers are provided in the Key Resources Table and in Table S4.
- The complete code used to compute the frequencies of the VH1–18 QxxV class in human IgM repertoires has been deposited in Zenodo and is publicly available from the date of publication. DOI is listed in the key resources table.
- Any additional information required to reanalyze the data reported in this paper is available from the lead contact upon request

### EXPERIMENTAL MODELS AND PARTICIPANT DETAILS

#### Generation of 09–1B12-UCA knock-in (KI) mice

09–1B12-UCA KI mice were generated following published protocols^39,40^. In brief, the targeting vector 4E10^78^ was modified by the incorporation of human rearranged 09–1B12-UCA KI VDJ (heavy chain construct) or VJ (light chain construct) sequences downstream of the promoter region and by elongation of the 5’ and 3’ homology regions using the Gibson assembly method (NEB). The targeting vector DNA was confirmed by Sanger sequencing (Eton Bioscience Inc.). Next, fertilized mouse oocytes were microinjected with a donor plasmid containing the pre-rearranged 09–1B12-UCA IGH with the mouse VHJ558 promoter, or the pre-rearranged 09–1B12-UCA IGK with the mouse Vk4–53 promoter (200 ng/mL); two pair of single-guide RNAs (sgRNAs, 25 ng/mL) targeting either the H or the k locus; and AltR-Cas9 protein (50 ng/mL) and injection buffer ^39^. The nucleotide sequence for 09–1B12-UCA IGH and IGK was from Corbett et al. ^37^. Following culture, resulting zygotes were implanted into the uteri of pseudopregnant surrogate C57Bl/6J mothers. F0-mice from the 09–1B12-UCA KI mouse (CD45.2^+/+^) colony were bred at the animal facility of the Gene Modification Facility (Harvard University) and breeding for colony expansion and experimental procedures was subsequently performed at the Ragon Institute of Mass General, MIT, and Harvard. Ear or tail snips from 09–1B12UCA KI mice were genotyped by TaqMan assay under a fee for service agreement (TransnetYX). TaqMan probes for the genotyping assay were developed by TransnetYX.

All experiments were performed under the approval by the Institutional Animal Care and Use Committee (IACUC) of Harvard University and the Massachusetts General Hospital (MGH) (Animal Study Protocols 2016N000022 and 2016N000286, 2014N000252) and conducted in accordance with the regulations of the Association for Assessment and Accreditation of Laboratory Animal Care International (AAALAC). Both male and female animals were used at 8–12 weeks of age. The light cycles in the animal room were set on a 12 hour light cycle [7AM-7PM (ON) 7PM-7AM (OFF)]. The temperature range for the room was 68 – 73 degrees Fahrenheit and the humidity index was from 30% – 70%. The feed was replaced every two weeks with fresh pelleted ration (Prolab Isopro RMH 3000), concomitant with changing fresh bedding in the cage. The cages were also inspected daily and additional pellets were added if food was low or empty.

### METHOD DETAILS

#### Adoptive transfer

For experiments male B6.SJL-*Ptprc*^a^*pepc*^b^/BoyJ mice (CD45.1^+/+^) 8–12 weeks of age were purchased from The Jackson Laboratory (Bar Harbor, ME). CD45.2^+^ B cells from male or female 09–1B12-UCA KI mice (*H*^09–1B12-UCA/WT^, *κ*^09−1B12-UCA/WT^) were enriched using the Pan B Cell Isolation Kit II (Miltenyi Biotec), counted, diluted to desired cell numbers in PBS and adoptively transferred into CD45.1^+^ recipient mice as reported previously^43^. Ages of the recipient animals ranged from 8–10 weeks.

#### Recombinant HA antigens and B cell probes

Recombinant H3 and H7 trimer ectodomains from A/Perth/16/2009 and A/Shanghai/02/2013, along with ferritin nanoparticle display of their trimeric stem domains (H3ssF and H7ssF) were affinity purified following expression in Expi293 cells according to established methodology^37,55,60,79^. The Expi293 cells were transfected with expression vector using the ExpiFectamine^™^ 293 Transfection Kit (Thermofisher), which supplies the transfection reagent and enhancer solution. Five days after transfection, the culture supernatants were harvested, filtered (VacuCap 8/0.2 μm filters, Pall Corporation) and buffer exchanged into PBS using a tangential flow filtration system [Pall Corporation; T-Series Centramate cassettes with Omega PES membrane 10 kDa (Cytiva, OS010T12)]. For HA trimers, the buffer exchanged supernatant was equilibrated with Ni Sepharose resin (GE Healthcare), whereas *Erythrina cristagalli* Gel-ECA-Immobilized Lectin (EY Laboratories) was mixed with the buffer exchanged supernatant containing H3ssF or H7ssF. Ni Sepharose was washed with 20 mM imidazole and the HA trimers subsequently eluted with 500 mM imidazole. For H3ssF and H7ssF, the resin was washed with PBS and the HA nanoparticles were eluted with 0.2 M lactose. All proteins were further purified by size exclusion chromatography (SEC) (AKTA pure protein purification system, Cytiva): HA trimers were resolved on a Superdex increase 200 10/300 column (Cytiva) and the stem nanoparticles were separated using a Superose 6 10/300 column (Cytiva). These same expression and purification procedures were applied to central stem epitope KO versions of the HA trimers and HA nanoparticles (containing N-linked glycan at 45_HA2_) and also for the recombinant H1 and H5 trimers used in this study (A/Michigan/45*/*2015, A/California/07/2009, A/Indonesia/05/2005). The HA trimers used in this study also contained the Y98F mutation in the RBS to prevent binding to sialyl oligosaccharide and foldon avi his sequence for trimerization/site specific biotinylation/affinity purification^28,55,60,80,81^.

B cell flow cytometry probes for H3ssF, H3ssF-KO, H7ssF, H7ssF-KO (central stem epitope KO = insertion of N-linked glycan at 45_HA2_) were generated by fluorescently labelled using amine reactive labeling kits (H3ssF-AF594, H7ssF-AF488, H7ssF-KO -AF647 + H3ssF-KO-AF647), as per our established procedure for ferritin nanoparticle-based B cell probes^28^. In some applications, HA trimers were avi-tagged and biotinylated at this site using the enzyme BirA and then flow probes (H3-APC and H7-AF488) were generated by adding fluorescent SA conjugates in five sequential increments (final molar ratio of HA to streptavidin label was 4:1) so as to saturate the site^60^.

For structural studies, HA trimers were produced following transfection into Expi293F GnTI^−^ (Gibco, #A39240) using ExpiFectamine (Gibco, #A14524). The proteins were purified over TALON metal affinity resin (Takara, #635653) and Superdex increase 200 10/300 column (Cytiva) on an AKTA Pure (Cytiva). C-terminal tags were cleaved with HRV 3C protease (Thermo Scientific, #88946) which was removed by TALON resin and purification over the S200 column.

#### B cell flow cytometry, FACS and sequencing to characterize 09–1B12-UCA KI mice (*H*^09–1B12-UCA/WT^, *κ*^09–1B12-UCA/WT^).

Cells from bone marrows (femurs and tibia) were isolated, filtered and resuspended in ACK lysis buffer to remove erythrocytes. Whole spleens were mechanically dissociated to generate single-cell suspensions and resuspended in ACK lysis buffer. Bone marrow cells and splenocytes were then separately resuspended in FACS buffer (2% FBS/PBS), Fc-blocked (clone 2.4G2, BD Biosciences) and stained for viability with Live/Dead Blue (Thermo Fisher Scientific) for 20 min at 4°C. For flow analysis and FACS, we applied H3ssF-AF594, H7ssF-AF488, H7ssF-KO-AF647, and H3ssF-KO-AF647 (0.25μg of each probe / 100 μl staining mix) along with the following fluorescent antibodies (1:200 final dilution): CD4-APCeF780, CD8-APC-eF780, Gr-1-APC-eF780, F4/80-APC-eF780, B220-PerCP Cy5.5, IgD-BV421 and IgM-BUV395. To measure B cell development, different fluorescent antibody panels were applied to splenocytes [TCRβ-AF700, B220-PerCP Cy5.5, CD24-PE, CD21-FITC, CD23-APC, CD8a-APC-e780, CD4-BV510, IgM-BV421 and IgD-PE Cy7] and bone marrow cells [B220-FITC, TCRb-AF700, CD43-APC, CD21-APC Cy7, BP-1-PE, CD24-BV421, IgD-PE Cy7, IgM-BUV395] at 1:200 final dilution. Flow cytometry was performed using a BD LSRFortessa (BD Biosciences) and the data were analyzed using FlowJo software (Tree Star). For single cell FACS, mature B cells gated as lymphocytes^+^/singlets^+^/Live-Dead^−^/dump^−^ (anti-mouse CD4, CD8, Ly-6G, F4/80)/ B220^+^/IgD^+^/IgM^+^ and antigen-specific CD45.2 B cells gated as lymphocytes^+^/ singlets^+^/ Live-Dead^−^/dump^−^ (anti-mouse CD4, CD8, Ly-6G, F4/80) /B220^+^/IgD^+^/IgM^+^/H3ssF^+^/H7ssF^+^/H3ssF-KO^−^/H7ssF-KO^−^ were sorted (BD FACS Aria II instrument with 70 μm nozzle) into 96-well PCR plates, and then rapidly frozen on dry ice and stored at −80°C.

Sorted single-cell suspensions were encapsulated and converted into several DNA libraries following the 10x Next GEM Single cell 5’ protocol (10x Genomics). Briefly, single cells were isolated with Gel Bead-In-EMulsions (GEMs) using the Chromium controller provided by 10x Genomics, resulting in uniquely barcoded transcriptome for each individual cell. After initial cDNA amplification and conversion to dsDNA, individual sequencing libraries were generated for gene expression, VDJ repertoires and hashtag oligos^82^. Library quality was analyzed using a Tapestation 4200 (Agilent). Libraries were pooled at a ratio based on depth requirements established by 10x Genomics and subsequently sequenced using a Nextseq2000 sequencer (llumina). Raw base call files generated by sequencing were demultiplexed, aligned and aggregated using the pipeline offered as part of Cell Ranger (10x Genomics). For VDJ repertoire analysis, immunoglobulin v genes were determined by Cell Ranger. Heavy and Light chains were subsequently paired based on 10x barcodes using a custom R script after doublet determination and removal using hashtag antibody sequences and the Seurat R package (https://satijalab.org/seurat/). Chord plots were produced in R circlize version 0.4.15 (https://cran.r-project.org/web/packages/circlize/index.html) package.

#### VH1–18-QxxV bnAb precursor frequency in humans

To compare VH1–18 QxxV bnAb precursor frequency within our adoptive transfer model to the natural value in humans, we measured this value in publicly available IgM BCR repertoires from n=10 human subjects sequenced to high depth^41^. From this dataset, we considered only those antibody sequences annotated as encoding productive IgM heavy chains. The complete code used to compute the frequencies of the VH1–18 QxxV class in these repertoires is available at https://www.doi.org/10.5281/zenodo.10800716. The Bash, R, and Python languages were used, as well as tidyverse ^83^ and pandas software packages^84^.

#### Sequential (2x) immunization of humanized mice and viral challenge

One day after adoptive transfer of CD45.2 B cells from 09–1B12-UCA KI mice, the recipient CD45.1 mice were given an intraperitoneal injection of 50 μg of H3ssF within 100 ml of inoculum containing 50% w/v Sigma adjuvant (Sigma, Cat# S6322; also known as Ribi), or Sigma adjuvant-only. We also included a CD45.1 mouse group that received H3ssF, but no UCA cells were present. Mice were boosted 42 days after the initial immunization (H3ssF + Sigma adjuvant for the mice primed with H3ssF; or Sigma adjuvant-only for the non-immunogen group). Fourteen days after the boost (day 56), the mice were intranasally infected with 100% lethal doses of either: subtype-matched H3N2 X-31 (BEI Resources cat# NR-3483) (10^8^ TCID_50_/ml); or group-unmatched, mouse-adapted H1N1 A/California/07/2009 (maA/Cal/09)^75,76^ (10^4^ TCID_50_/ml). Mice were monitored each day for 21 days for survival and body weight loss. The humane endpoint was set at 20% weight loss. Both viruses were cultured in MDCK cells and quantified by TCID_50_ in MDCK cells^85^. X-31 was obtained from BEI Resources and maA/Cal/09 was kindly provided by Sabra Klein and Andrew Pekosz, John Hopkins University.

#### Single shot immunization and lineage tracking within B cell germinal centers

One day after adoptive transfer of CD45.2 B cells from 09–1B12-UCA KI mice, the recipient CD45.1 animals were immunized intraperitoneally with either: 50 μg H3ssF; 50 μg H7ssF; or 50 μg of ferritin-only nanoparticle. In all cases, a 200 μl inoculum containing 50% w/v Sigma adjuvant was deployed. On days 8, 15, and 28, whole spleens were mechanically dissociated to generate single-cell suspensions. ACK lysis buffer was used to remove red blood cells and splenocytes were then resuspended in FACS buffer (2% FBS/PBS), Fc-blocked (clone 2.4G2, BD Biosciences) and stained for viability with Live/Dead Blue (Thermo Fisher Scientific) for 20 min at 4°C. For flow staining we applied H3ssF-A594, H7ssF-A488, H7ssF-KO-A647, H3ssF-KO-A647 (or trimer probes: H3-APC and H7-AF488) at 0.25μg of probe/100 μl staining mix along with following fluorescent antibodies (1:200 final dilution): CD4-APCeF780, CD8-APC-eF780, Gr-1-APC-eF780, F4/80-APC-eF780, B220-BV605, CD95-PE-Cy7, CD38-BV510, CD45.1-PerCPCy5.5, CD45.2-BV785. Cells were acquired by a BD LSR Fortessa (BD Biosciences) and the data were analyzed using FlowJo software (Tree Star). Single GC CD45.2 B cells with specificity to the central stem [lymphocytes^+^/singlets^+^/Live-Dead^−^/dump^−^ (anti-mouse CD4, CD8, Ly-6G, F4/80) /B220^+^/CD95^+/^CD38^−^/CD45.1^−^/CD45.2^+^/Ag^+^(H3ssF-AF594^+^/H7-AF488^+^/H3ssF-KO^−^/H7ssF-KO^−^)] were also were sorted using a BD FACS Aria II instrument (BD Biosciences) with 70 μm nozzle into 96-well plates containing RLT lysis buffer supplemented with 1% beta-mercaptoethanol (BME). The sorted cells were promptly frozen and stored at −80°C for future analysis.

#### BCR sequencing of GC B cells

Single cell BCR libraries were generated from products of whole transcriptome amplification (WTA) using the Smart-Seq2 protocol^86^. The WTA product from each single cell reaction were first subjected to two 0.8x (v/v) SPRI bead-based cleanups followed by cDNA quantification/normalization. BCR sequences from heavy and light chains were enriched from single B cells using a V region and J region specific primer set in which the primers were also attached to Illumina P7 (V region) and P5 sequences (J region) (final concentration: 0.5 μM each) (Table S5). After amplification, a 0.8x (v/v) SPRI cleanup was performed, and we quantified and normalized the amplicons to 0.2–0.5 ng/μL. Within a subsequent step-out PCR [Kapa HiFi HotStart ReadyMix; Kapa Biosystems], we added cellular barcodes and Illumina sequencing adapters (based on Nextera XT Index Adapters, Illumina Inc.) to each single cell-amplified heavy and light chain, as we have performed previously^28,55,61^. After a 0.8x (v/v) SPRI cleanup, the heavy chain and light chain products were pooled and sequenced using paired end 250×250 reads and 8×8 index reads on an Illumina MiSeq System [MiSeq Reagent Kit v2 (500-cycle)]. The BCR heavy and light chains were paired and reconstructed using PandaSeq^87^ and aligned against the human IMGT database^88,89^ with PCR/sequencing error correction using MigMAP (a wrapper for IgBlast: https://github.com/mikessh/migmap). Consensus V-chain and L/k-chain sequences for each single cell was performed by collapsing all reads with the same CDR3 sequence and then identifying the top heavy and light chain sequences based on frequency. Any heavy or light chain sequence with fewer than 15 reads or a frequency less than twice that of the next sequence of the same chain was considered without consensus. Phylogenetic trees to visualize relatedness were generated from the heavy chain nucleotide sequences using the maximum likelihood method with the Tamura-Nei model in MEGA11 software^90,91^. Heavy chain and light chain sequences from 09–1B12-UCA were used as the baseline for tree construction. Sequence logos were generated using WebLogo (https://weblogo.berkeley.edu)^92^.

#### Recombinant Monoclonal Antibodies and Fabs

The variable sequences from BCR heavy and light chains were synthesized as gene blocks by GenScript. The sequences were cloned into human IgG1 heavy chain and light chain expression plasmids^28,55,61^. The antibodies were transfected into Expi293 cells, as described for the recombinant HA trimers and HA nanoparticles, except that we harvested the supernatant after six days of culture. Following filtration of the supernatant (VacuCap 8/0.2 μm filters, Pall Corporation) the material was equilibrated with Protein G Sepharose resin. After washing with PBS, the IgG was eluted with IgG elution buffer (Pierce, Cat# 21028) into 1M Tris, pH 8. The eluate was further purified and buffer exchanged to PBS using SEC with a Superdex 200 10/300 column (GE Healthcare). Additional monoclonal antibodies were expressed and purified in the same way: 09–1B12-UCA, 09–1B12, 21–1A01-UCA, 21–1A01; 05–2A09-UCA, 05–2A09, 05–2D04-UCA, 27–1D08-UCA, 06–1F04-UCA^24,37^; 09–1B12-UCA + N55T, 21–1A01-UCA + N55T, 05–2A09-UCA + N55T, 05–2D04-UCA + N55T, 27–1D08-UCA + N55T, 06–1F04-UCA + N55T; CR6261^93,94^; VRC01^95^. The HC and LC nucleotide sequences of 09–1B12-UCA was from Corbett et al.^37^, the other UCAs were inferred by gHgL reversion of the mature antibody sequence. For structural studies, antibody Fab fragments were produced by transfection into HEK293F (Gibco, #R79007) using PEI at a 3:1 ratio, followed by expression and his tag-based affinity purification and cleavage of the tag with HRV 3C protease, as described earlier for HA trimers.

#### Neutralization Assay

Microneutralization assays were performed according to Creanga et al.^46^. HEK293T, HEK293T-PB1 (constitutively expressing A/WSN/1933 PB1), MDCK-SIAT1-PB1, MDCK-SIAT1-H5 (constitutively expressing A/Vietnam/1203/2005 HA), and MDCK-SIAT1-H7 (constitutively expressing A/Shanghai/02/2013 HA) were maintained in DMEM (Gibco, #11965084) supplemented with 10% heat-inactivated FBS (Peak Serum) and 100 U of penicillin/streptomycin (Gibco, #15140163)^46^. Media for MDCK-SIAT1-PB1, -H5, and -H7 cells was further supplemented with 1 mg/ml geneticin (Gibco, #10131035) and 0.25 μg/ml puromycin (Gibco, #A1113803). Prior to infection, cells were seeded in “flu media” composed of Opti-MEM (Gibco, #31985088) supplemented with 0.3% BSA (Sigma, #A9418), 100 U of penicillin/streptomycin (Gibco), 0.1 mg/ml of CaCl_2_, and 0.01% heat-inactivated FBS (Peak Serum).

Influenza reporter viruses were generated as described previously^46^. Briefly, H1N1 and H3N2 viruses were generated using plasmids encoding bidirectional cassettes for PB2, PA, NP, M, and NS from A/WSN/1933^96^, tdKatushka2 containing PB1 packing sequences, a plasmid encoding human TMPRSS2, and HA and NA segments flanked with non-coding regions from A/WSN/1933 (for H1N1 viruses) or A/Netherlands/009/2010 (for H3N2 viruses). Plasmids were transfected into HEK293T-PB1 cells in a 6-well plate using TransIT-LT1 (MirusBio, #2306). After three days, the supernatant was clarified by centrifugation at 800 ×g for 5 minutes and added to a monolayer of MDCK-SIAT1-PB1 cells in a 6-well plate. Two to three days later when cytopathic effect was evident, the supernatant was clarified and added to a monolayer of MDCK-SIAT1-PB1 cells in T75 or T150 flasks. Two to three days later, the supernatant was clarified, and aliquots of viral stocks were frozen at −80 °C. To generate H5N1 and H7N9 viruses, the internal genes (PB1, PB2, PA, NP, M, NS) from A/Puerto Rico/8/1934 were used with tdKatushka2 bearing the HA packing sequences from A/Puerto Rico/8/1934. For H5N1 and H7N9 viruses, a plasmid expressing H5 HA (A/Vietnam/1203/2004) or H7 HA (A/Shanghai/02/2013) were additionally transfected into HEK293T cells. H5N1 and H7N9 viruses were passaged and propagated as above in MDCK-SIAT1-HA cells constitutively expressing H5 or H7 HA.

One day prior to titering, MDCK-SIAT-PB1 (or -HA) cells were seeded in 96-well plates at 20,000–30,000 cells per well (CellVis, #P96–1.5P). The next day, viral stocks were thawed and diluted 2-fold 23 times in quadruplicate. The virus dilutions were then mixed with an equal volume of flu media additionally supplemented with 2 μg/ml of TPCK-treated trypsin (Sigma, #T1426). Virus was incubated at 37 °C / 5% CO_2_ for 1 hour before removing flu media from MDCK-SIAT1-PB1 (or -HA) cells and adding 100 μl of the virus. Approximately 18–20 hours later, the cells in each well were imaged on a CellDiscoverer7 (Zeiss) instrument and fluorescent cells were counted. The cell counts were plotted, and a sigmoidal curve was fitted in GraphPad Prism. The dilution that corresponded to the half maximal positive cell count was used for subsequent neutralization assays.

For microneutralization measurements, the viruses were diluted in flu media with 2 μg/ml of TPCK-treated trypsin and antibodies were diluted in a four-fold series in flu media. The virus and antibody dilutions were mixed in equal volumes in a 96-well plate and incubated at 37 °C / 5% CO_2_ for 1 hour. The flu media was then removed from the MDCK-SIAT1-PB1 (or -HA) cells and the antibody/virus mixture was added. Approximately 18–20 hours later, the cells were counted as above. Each plate contained ten virus only control wells and two cell only controls. Each value had the cell only control background subtracted and was normalized to the average of the virus only controls. The percent neutralization was plotted and fitted with a sigmoidal curve (GraphPad Prism) to determine the half maximal inhibitory concentration (IC_50_). Each neutralization assay was run in duplicate.

#### ELISA

To assess the IgG titers of immunized mice, 200 ng per well of either H3ssF, H7ssF, or their respective epitope KO versions, were pre-coated in 96-well plates at 4°C overnight. After incubation with blocking buffer (3% BSA in PBS + 0.01% tween 20) for 2 h at RT, 3-fold serial diluted sera (7 dilutions) with starting dilution 1:100 from priming or boosting mice were incubated with pre-coated protein for 2 h at RT. Wells were washed and incubated with Alkaline Phosphatase AffiniPure Goat Anti-Mouse IgG (Jackson ImmunoResearch, #115–055-071) at 1:1,000 in PBS with 0.5% BSA for 1 h at RT. P-Nitrophenyl phosphate (Sigma, # N2770) dissolved in ddH2O (50 μL/well, RT, 25 min) was used for detection. Absorbance at 405 nm was determined with a plate reader (Synergy Neo2, BioTek). ELISA curves were calculated and analyzed using GraphPad Prism 9.5.1 (GraphPad).

#### Immunization of humanized mice and serum processing for CryoEMPEM

One day after adoptive transfer of CD45.2 B cells from 09–1B12-UCA KI mice, the recipient CD45.1 mice were immunized intraperitoneally with either 50 μg H3ssF or 50 μg H7ssF; each delivered in a 100 ml inoculum containing 50% w/v Sigma adjuvant. The mice were boosted again at 42 days with these same inoculums and immune sera were obtained at 15 and 28 days post-boost. These sera were then processed for cryoEMPEM analysis^48^. Briefly, Serum IgG was purified from pooled sera (in the same group) with Protein G resin and subsequently digested with papain. Digested polyclonal Fabs were purified over Superdex 200 Increase column (Cytiva).

#### CryoEM

For cryoEM, HA (30 μg) was incubated with the purified monoclonal Fab (40 ug) or polyclonal Fabs (0.5–1 mg) overnight at 4 °C. The complex was then purified over Superdex 200 Increase column (GE Healthcare) and concentrated to ~0.7 mg/ml. Next, 3 μL of the complex was mixed with 0.5 μL 0.7% (w/v) Octyl-beta-Glucoside (OBG) before deposition onto glow-discharged 1.2/1.3 Cu 300 grids (EMS), directly preceding the deposition in a Vitrobot (Thermo Fisher Scientific) with following settings: 4°C, 100% humidity, 0 s wait time, 4.5–6 s blot time, blot force 1. Once sample was deposited, the grids were blotted and plunged into liquid to immobilize the particles in vitreous ice. The micrographs were collected with EPU image acquisition software at a nominal magnification of ×190,000 with a TFS Falcon 4 detector mounted on a Glacios set to 200 kV set to counting mode, with a total exposure dose of ~50 e^−^/Å^2^. Micrographs were collected in CryoSPARC Live and followed by gain reference correction, motion correction, defocus estimation, and micrograph curation (Table S1). Template Picker was used to pick particles, which were then extracted and 2D-classified in cryoSPARC. The particles in selected 2D classes were further cleaned up by Heterogenous refinement using C1 symmetry and 3D Variability with symmetry expanded particles. Final maps were made by homogeneous refinements with tight masks on HA and Fab on one protomer.

For model building, the initial models were generated from PDBID: 3LZG (H1), 4KVN (H3), 4N5J (H7), and 5KAN (V1–18 Fab), and docked into cryoEM map using UCSF ChimeraX^97^. Coot 0.9.8^98^, Phenix^99^, and Rosetta^100^ were used for model building and refinement (Table S1). The final models and cryoEM maps have been deposited in PDB (8UT3, 8UT4, 8UT5, 8UT6, 8UT7, 8UT8, 8UT9) and EMDB (EMD-42528, EMD-42529, EMD-42530, EMD-42531, EMD-42532, EMD-42533, EMD-42534, EMD-42535, EMD-42536).

#### X-ray crystallography

09–1B12 Fab and H3 Perth/2009 HA were complexed at a 4:1 molar ratio for 20 minutes at room temperature prior to concentration and purification over an S200 column (Cytiva). Crystals were generated by the hanging drop method over 0.1 M sodium citrate tribasic dihydrate (pH 5.0), 10% v/v 2-propanol, and 10% PEG 10k (Hampton Research, #HR2–084) for ~7 days at 18 °C using 96-well plates (Greiner, #655101) with ViewDrop II seals (sptlabtech, #4150–05600). Crystals were harvested after adding 1 μl of 12% (+/−)-2-methyl-2,4-pentanediol (MPD) in the crystallization solution and plunged into liquid nitrogen to freeze.

X-ray diffraction data was collected at beam line 24-ID-E (Advanced Photon Source) and processed with XDSGUI (https://strucbio.biologie.uni-konstanz.de/xdswiki/index.php/XDSGUI). The work used NE-CAT beamlines (GM124165), a Pilatus detector (RR029205), and an Eiger detector (OD021527) at the APS (DE-AC02–06CH11357). The structure was initially solved by molecular replacement using the HA protomer from PDB 4KVN, 16.a.26 variable heavy chain with the CDRH3 removed (from PDB 5K9Q), and a model of the variable light chain with the CDRL3 removed^101^. PHENIX^102^ was used to initially refine the coordinates and B factors prior to manual model building in COOT^103^. Final refinement additionally included Translation Libration Screw refinements and Ramachandran restraints (Table S2). The final model was deposited to the Protein Data Bank (8UWA).

#### Structure Modelling and MD simulations

To characterize the interaction profiles of the UCA/09–1B12 antibodies in complex with H1, H3 and H7 we performed molecular dynamics simulations of these antibody complexes, respectively. As starting structures for our simulations, we used the available cryo-EM structures, presented in this study. For all these antibody-antigen complexes we performed each three replicas of 1μs classical molecular dynamics simulations. Furthermore, we also performed simulations of the free variable domains (Fvs), namely 09–1B12-UCA, 09–1B12-UCA + N55T and 09–1B12, to characterize the effect of the mutations on the dynamic properties of the Fvs.

The starting structures for our simulations were prepared in MOE using the Protonate3D tool^104,105^. With the help tool of the Amber Tools20 package^106^, we explicitly bonded all existing disulfide bridges and placed the antibody-antigen complexes into cubic water boxes of TIP3P water molecules with a minimum wall distance to the protein of 12 Å^107^. Parameters for all antibody-antigen and free Fv simulations were derived from the CHARMM36m ^108^. To neutralize the charges, we used uniform background charges^106,109^. Each system was equilibrated using a multistep equilibration protocol^110^. Molecular dynamics simulations were performed using pmemd.cuda in an NpT ensemble to be as close to the experimental conditions as possible and to obtain the correct density distributions of both protein and water^111^. Bonds involving hydrogen atoms were restrained by applying the SHAKE algorithm^112^, allowing a timestep of 2.0 fs. Atmospheric pressure of the system was preserved by weak coupling to an external bath using the Berendsen algorithm^113^. The Langevin thermostat was used to maintain the temperature at 300K during simulations^114,115^.

To calculate contacts of the antibody-antigen complexes, we used the GetContacts software (https://getcontacts.github.io/).This tool can compute interactions within one protein structure, but also between different protein interfaces and allows to monitor the evolution of contacts during the simulation. The contacts are defined based on the default geometrical criteria provided by GetContacts.

#### Biolayer interferometry (BLI)

For BLI, Fabs were produced by digestion of recombinant IgGs with Lys-C endoproteinase (New England Biolabs Cat. # P8109S)^28,55,61^. The reaction was in PBS containing 1 mM EDTA, with a ratio of 5 mg of Lys-C per milligram of IgG. After incubating for 12 hours at room temperature, the reaction was stopped by addition of 1x complete protease inhibitor cocktail (Roche, Cat # 11697498001) and Protein A/G-agarose was applied to remove any uncleaved IgG. The resin was washed with PBS, and the supernatant was concentrated using Amicon Ultra concentrators with a 10 kDa cutoff. The Fabs were further purified by SEC, using a Superdex 200 10/300 column (GE Healthcare). BLI was performed using the Personal Assay BLItz System (Fortebio). Following avi-tag biotinylation (see earlier), the biotinylated forms of HA trimers spanning group 1 and group 2 diversity (A/Perth/16/2009, A/Shanghai/02/2013, A/*Michigan*/45/*2015,* A/*California*/07/*2009*, A/Indonesia/05/2005) were immobilized onto streptavidin biosensors (Sartorius, Cat#18–5019). After establishing a baseline in kinetic buffer (PBS containing 0.02% Tween20, 0.1% BSA), the Fabs were introduced at 0.625 mM, 1.25 mM, 2.5 mM, and 5 mM, with 120 seconds of association and 120 seconds of dissociation. The equilibrium dissociation constant (KD) values were calculated by fitting a 1:1 binding isotherm using software provided by the vendor^77^.

#### Passive transfer of monoclonal antibodies and viral challenge

Monoclonal antibodies were infused at 5 mg/kg intraperitoneally into WT C57Bl/6J mice^24^. Two hours later, the mice were intranasally infected with 100% lethal doses of either: subtype-matched H3N2 X-31 (BEI Resources cat# NR-3483) (10^8^ TCID_50_/ml); or group-unmatched, mouse-adapted H1N1 A/California/07/2009 (maA/Cal/09)^75,76^ (10^4^ TCID_50_/ml). The mice were monitored each day for 21 days for survival and body weight loss. The humane endpoint was set at 20% weight loss, as described above.

### QUANTIFICATION AND STATISTICAL ANALYSES

All statistical analysis were conducted using Prism 9.01 (GraphPad). Sample sizes and statistical tests are indicated in the figure legends. Data were considered statistically significant at P<0.05.

## Supplementary Material

1

2

## Figures and Tables

**Key resources table:** 

REAGENT or RESOURCE	SOURCE	IDENTIFIER
Antibodies		
PerCP/Cy5.5 anti-mouse CD45.1 Antibody	Biolegend	Cat#110728; RRID:AB_893348
PE-Cy^™^7 Hamster Anti-Mouse CD95	BD Biosciences	Cat#557653; RRID:AB_396768
Brillant Violet 785^™^ anti-mouse CD45.2	Biolegend	Cat#109839; RRID:AB_2562604
Anti-mouse CD38, BV510	BD Biosciences	Cat#740129; RRID:AB_2739886
BV711 Rat Anti-Mouse Ig, λ1, λ2 & λ3 Light Chain	BD Biosciences	Cat#744527; RRID:AB_2742301
BUV395 Rat Anti-Mouse Ig, κ light chain	BD Biosciences	Cat#742839; RRID:AB_2741090
BV421 Rat Anti-Mouse IgM	BD Biosciences	Cat#743323; RRID:AB_2741424
PE/Cy7 anti-mouse IgD Antibody	Biolegend	Cat#405720; RRID:AB_2561875
PE anti-mouse CD45.2 Antibody	Biolegend	Cat#109808; RRID:AB_313445
Anti-mouse CD38 Alexa700	Invitrogen	Cat#56-0381-82
BV786 Rat Anti-Mouse IgD	BD Biosciences	Cat#563618; RRID:AB_2738322
Alexa Fluor^®^ 700 anti-mouse IgD Antibody	BD Biosciences	Cat#405730
BV605 Rat anti-mouse CD45R/B220	BD Biosciences	Cat#563708; RRID:AB_2738383
PE anti-mouse CD138 (Syndecan-1) Antibody	Biolegend	Cat#142504; RRID:AB_10915989
BV510 Rat Anti-Mouse IgD	BD Biosciences	Cat#563110; RRID:AB_2737003
Brilliant Violet 421^™^ anti-mouse IgD Antibody	Biolegend	Cat#405725; RRID:AB_2562743
BV510 Rat Anti-Mouse B220/CD45R Clone RA3-6B2	BD Biosciences	Cat#563103; RRID:AB_2738007
BV421 Rat Anti-Mouse IgG1	BD Biosciences	Cat#562580; RRID:AB_2737664
BUV395 Rat Anti-Mouse IgM	BD Biosciences	Cat#743329; RRID:AB_2741430
CD4 Monoclonal Antibody (GK1.5), APC-eFluor 780	Invitrogen	Cat#47-0042-80
CD8a Monoclonal Antibody (53-6.7), APC-eFluor 780	Invitrogen	Cat#47-0081-80
F4/80 Monoclonal Antibody (BM8), APC-eFluor 780	Invitrogen	Cat#47-4801-80
Ly-6G Monoclonal Antibody (1A8-Ly6g), APC-eFluor 780	Invitrogen	Cat#47-5931-80
FITC anti-mouse/human CD45R/B220 Antibody	Biolegend	Cat#103206; RRID:AB_312991
APC anti-mouse CD43 Antibody	Biolegend	Cat#143208; RRID:AB_11149685
APC/Cy7 anti-mouse CD21/CD35 (CR2/CR1) Antibody	Biolegend	Cat#123418; RRID:AB_1953275
BV421 Rat Anti-Mouse CD24	BD Biosciences	Cat#562563; RRID:AB_2737002
Anti-mouse CD249 (BP1) FITC	Thermo	Cat#11-5891-82; RRID:AB_465295
Alexa Fluor 700 anti-mouse TCRb chain	BioLegend	Cat#109224; RRID:AB_1027654
FITC anti-mouse CD21/CD35 (CR2/CR1) Antibody	BioLegend	Cat#123408; RRID:AB_940403
PerCP/Cy5.5 anti-mouse/human CD45R/B220 Antibody	BioLegend	Cat#103236; RRID:AB_893354
APC anti-mouse CD23 Antibody	BioLegend	Cat#101620; RRID:AB_2563438
PE anti-mouse CD24 Antibody	BioLegend	Cat#101808; RRID:AB_312840
Anti Mouse CD4 BV510	BioLegend	Cat#100559; RRID:AB_2562608
BV421 Rat Anti-Mouse IgM	BD Biosciences	Cat#743323; RRID:AB_2741424
Alkaline Phosphatase AffiniPure Goat Anti-Mouse IgG, Fcγ Fragment Specific	Jackson ImmunoResearch	Cat#115-055-071
Purified Rat Anti-Mouse CD16/CD32 (Mouse BD Fc Block^™^)	BD Biosciences	Cat#553142; RRID:AB_394657
Alexa Fluor^®^ 488 anti-mouse CD45.2 Antibody	Biolegend	Cat#109816; RRID:AB_492868
Alexa Fluor^®^ 594 anti-mouse CD3 Antibody	Biolegend	Cat#100240; RRID:AB_2563427
TotalSeq^™^-C0301 anti-mouse Hashtag 1 Antibody	Biolegend	Cat#155861; RRID:AB_2800693
TotalSeq^™^-C0302 anti-mouse Hashtag 2 Antibody	Biolegend	Cat#155863; RRID:AB_2800694
TotalSeq^™^-C0303 anti-mouse Hashtag 3 Antibody	Biolegend	Cat#155865; RRID:AB_2800695
TotalSeq^™^-C0304 anti-mouse Hashtag 4 Antibody	Biolegend	Cat#155867; RRID:AB_2800696
TotalSeq^™^-C0305 anti-mouse Hashtag 5 Antibody	Biolegend	Cat#155869; RRID:AB_2800697
TotalSeq^™^-C0306 anti-mouse Hashtag 6 Antibody	Biolegend	Cat#155871; RRID:AB_2819910
TotalSeq^™^-C0307 anti-mouse Hashtag 7 Antibody	Biolegend	Cat#155873; RRID:AB_2819911
TotalSeq^™^-C0308 anti-mouse Hashtag 8 Antibody	Biolegend	Cat#155875; RRID:AB_2819912
TotalSeq^™^-C0309 anti-mouse Hashtag 9 Antibody	Biolegend	Cat#155877; RRID:AB_2819913
TotalSeq^™^-C0310 anti-mouse Hashtag 10 Antibody	Biolegend	Cat#155879; RRID:AB_2819914
09-1B12-UCA	Produced in house (Corbett et al. 2019)	N/A
09-1B12-UCA + N55T	Produced in house	N/A
09-1B12	Produced in house (Andrews et al. 2017)	N/A
05-2A09-UCA	Produced in house	N/A
05-2A09-UCA + N55T	Produced in house	N/A
05-2A09	Produced in house (Andrews et al. 2017)	N/A
21-1A01-UCA	Produced in house	N/A
21-1A01-UCA- + N55T	Produced in house	N/A
21-1A01	Produced in house (Andrews et al. 2017)	N/A
05-2D04-UCA	Produced in house	N/A
05-2D04-UCA + N55T	Produced in house	N/A
05-2D04	Produced in house (Andrews et al. 2017)	N/A
27-1D08-UCA	Produced in house	N/A
27-1D08-UCA + N55T	Produced in house	N/A
27-1D08	Produced in house (Andrews et al. 2017)	N/A
O1	Produced in house	N/A
O2	Produced in house	N/A
VRC01	Produced in house	N/A
CR6261	Produced in house	N/A
Biological samples		
H3N2 X-31	BEI Resources	Cat#NR-3483
Mouse adapted H1N1 A/California/07/2009 (maA/Cal/09)	Sabra Klein and Andrew Pekosz John Hopkins University (Fink et al. 2018)	N/A
Influenza reporter viruses	Masaru Kanekiyo, NIH (Creanga et al. 2021)	N/A
Chemicals, peptides, and recombinant proteins		
Pan B Cell Isolation Kit II, mouse	Miltenyi Biotec	Cat#130-104-443
LIVE/DEAD^™^ Fixable Blue Dead Cell Stain Kit, for UV excitation	Thermo Fisher Scientific	Cat#L34962
CountBright^™^ Absolute Counting Beads, for flow cytometry	Thermo Fisher Scientific	Cat#C36950
SIGMAFAST^™^ p-Nitrophenyl phosphate Tablets	Sigma	Cat#N2770-50SET
NP40	Millipore	Cat#492016-100ML
UltraComp eBeads^™^ Compensation Beads	Thermo Fisher Scientific	Cat#01-2222-42
H3ssF	Produced in house (Corbett et al. 2019)	N/A
H7ssF	Produced in house (Corbett et al. 2019)	N/A
Invitrogen^™^ Molecular Probes^™^ DAPI (4’,6-Diamidino-2-Phenylindole, Dihydrochloride)	Thermo Fisher Scientific	Cat#D1306
Chromium Next GEM Single Cell 5’ Kit v2	10x Genomics	Cat#PN-1000263
Library Construction Kit	10x Genomics	Cat#PN-1000190
Chromium Single Cell Mouse BCR Amplification Kit	10x Genomics	Cat#PN-1000255
Chromium Next GEM Chip K Single Cell Kit	10x Genomics	Cat#PN-1000286
Dual Index Kit TT Set A	10x Genomics	Cat#PN-1000215
Dual Index Kit TN Set A	10x Genomics	Cat#PN-1000250
Penicillin/streptomycin	Gibco	Cat#15140163
Puromycin	Gibco	Cat#A1113803
Opti-MEM	Gibco	Cat#31985088
Bovine Serum Albumin	Sigma	Cat#A9418
TransIT-LT1	MirusBio	Cat#2306
Gibson assembly kit	NEB	Cat#E5510S
ExpiFectamine^™^ 293 Transfection Kit	Thermo Fisher Scientific	Cat#A14525
Ni Sepharose resin	GE Healthcare	N/A
A/Perth/16/2009 H3 trimer	Produced in house (Joyce et al. 2016)	N/A
A/Shanghai/02/2013 H7 trimer	Produced in house (Joyce et al. 2016)	N/A
A/Michigan/45/2015 H1 trimer	This paper	N/A
A/California/07/2009 H1 trimer	Produced in house (Sangesland et al. 2019)	N/A
A/Japan/305/1957 H2 trimer	This paper	
A/Indonesia/05/2005 H5 trimer	Produced in house (Whittle et al. 2014)	N/A
A/Perth/16/2009 H3 trimerDstem (KO, 45_HA2_)	This paper	N/A
A/Shanghai/02/2013 H7 trimerDstem (KO, 45_HA2_)	This paper	N/A
A/Michigan/45/2015 H1 trimerDstem (KO, 45_HA2_)	This paper	N/A
A/California/07/2009 H1 trimerDstem (KO, 45_HA2_)	This paper	N/A
A/Indonesia/05/2005 H5 trimerDstem (KO, 45_HA2_)	This paper	N/A
H3ssF-KO (45_HA2_)	This paper	N/A
H7ssF-KO (45_HA2_)	This paper	N/A
Sigma Adjuvant System	Sigma-Aldrich	Cat#S6322
Protein G Sepharose	GE Healthcare	Cat#17061802
KAPA HiFi HotStart ReadyMix	KAPA Biosystems	Cat#KK2602
Maxima H Minus Reverse Transcriptase	Thermo Fisher	Cat#EP0751
HotStarTaq Plus DNA Polymerase	QIAGEN	Cat#203603
Buffer RLT	QIAGEN	Cat#79216
BirA Biotin-Protein Ligase Bulk Reaction Kit	Avidity	Cat#Bulk BirA
Alexa Fluor 647 Protein Labeling Kit	Thermo Fisher	Cat#A20173
Alexa Fluor 488 Protein Labeling Kit	Thermo Fisher	Cat#A10235
Alexa Fluor 594 Protein Labeling Kit	Thermo Fisher	Cat#A10239
Alexa Fluor 546 Protein Labeling Kit	Thermo Fisher	Cat#A20183
MiSeq Reagent Kit, V2 500 cycles	Illumina	Cat#MS-102-2003
10% PEG 10k	Hampton Research	Cat#HR2-084
Lys-C endoproteinase	NEB	Cat#P8109S
Protease inhibitor cocktail (PIC)	Roche	Cat#11697498001
Deposited data		
VH1-18 heavy chain (HC) and light chain (LC) sequences (see also Data S1)	This paper	GenBank IDs: PP507169 - PP508172
X-ray structure of 09-1B12 bound to A/Perth/14/2009 H3N2 hemagglutinin	This paper	PDB ID: 8UWA
CryoEM structure of A/Perth/16/2009 H3 in complex with flu HA central stem VH1-18 antibody UCA6	This paper	PDB ID: 8UT3, EMDB ID: EMD-42528
CryoEM structure of A/Michigan/45/2015 H1 in complex with flu HA central stem VH1-18 antibody 09-1B12	This paper	PDB ID: 8UT4, EMDB ID: EMD-42529
CryoEM structure of A/Michigan/45/2015 H1 in complex with flu HA central stem VH1-18 antibody UCA6_N55T	This paper	PDB ID: 8UT5, EMDB ID: EMD-42530
CryoEM structure of A/Perth/16/2009 H3 in complex with polyclonal Fab from mice immunized with H3 stem nanoparticles-15 days post immunization	This paper	PDB ID: 8UT6, EMDB ID: EMD-42531
CryoEM structure of A/Perth/16/2009 H3 in complex with polyclonal Fab from mice immunized with H3 stem nanoparticles-28 days post immunization	This paper	PDB ID: 8UT7, EMDB ID: EMD-42532
CryoEM structure of A/Shanghai/1/2013 H7 in complex with polyclonal Fab from mice immunized with H7 stem nanoparticles-15 days post-immunization	This paper	PDB ID: 8UT8, EMDB ID: EMD-42533
CryoEM structure of A/Shanghai/1/2013 H7 in complex with polyclonal Fab from mice immunized with H7 stem nanoparticles-28 days post immunization	This paper	PDB ID: 8UT9, EMDB ID: EMD-42534
CryoEM structure of A/Perth/16/2009 H3	This paper	EMDB ID: EMD-42535
CryoEM map of A/Shanghai/1/2013 H7 HA	This paper	EMDB ID: EMD-42536
Code used to compute frequencies of the VH1-18 QxxV class in human IgM repertoires	This paper	https://www.doi.org/10.5281/zenodo.10800716
Experimental models: Cell lines		
Human: FreeStyle 293F	Thermo Fisher	Cat#R79007; RRID:CVCL_D603
Human: Expi293F	Thermo Fisher	Cat#A14527; RRID:CVCL_D615
Canine: MDCK	ATCC	Cat#CCL-34; RRID:CVCL_0422
Human: HEK293F	Gibco	Cat#R79007; RRID:CVCL_6642
MDCK-SIAT1-PB1	Produced in house (Creanga et al. 2021)	N/A
MDCK-SIAT1-H5	Produced in house (Creanga et al. 2021)	N/A
MDCK-SIAT1-H7	Produced in house (Creanga et al. 2021)	N/A
Expi293F GnTI^−^	Produced in house	N/A
HEK293T-PB1	Produced in house (Creanga et al. 2021)	N/A
Experimental models: Organisms/strains		
Mouse: B6.SJL-*Ptprc*^a^*pepc*^b^/BoyJ mice	The Jackson Laboratory	JAX: 002014; RRID:IMSR_JAX:002014
Mouse: C57BL/6	The Jackson Laboratory	JAX: 000664; RRID:IMSR_JAX:000664
Mouse: 09-1B12-UCA KI mouse (*H^09-1B12-UCA/WT^, k^09-1B12-UCA/WT^*)	This paper	N/A
Oligonucleotides		
TaqMan probes for genotyping	TransnetYX	N/A
Cocktails of IgG- and IgK-specific primers and thermocycling conditions described previously	von Boehmer et al. 2016	N/A
sgRNA	Lin et al. 2018; Wang et al. 2021	N/A
Primers for Single Cell BCR Amplification, see Table S4	This paper	N/A
Software and algorithms		
Flowjo 10.9.0	Treestar	https://www.flowjo.com/; RRID:SCR_008520
Prism 10.1.0	GraphPad	https://www.graphpad.com/; RRID:SCR_002798
IMGT/V-quest	Brochet et al. 2008; Giudicelli et al. 2011	http://www.imgt.org/IMGTindex/V-QUEST.php; RRID:SCR_010749
Geneious Prime	Biomatters	https://www.geneious.com/; RRID:SCR_010519
Cell Ranger	10x Genomics	https://support.10xgenomics.com/single-cell-gene-expression/software/pipelines/latest/what-is-cell-ranger; RRID:SCR_017344
WebLogo	Crooks et al., 2004	https://weblogo.berkeley.edu; RRID:SCR_010236
Seurat R package	Standard R Package	https://satijalab.org/seurat/; RRID:SCR_016341
R circlize 0.4.15	R package	https://cran.r-project.org/web/packages/circlize/index.html; RRID:SCR_002141
Bash 5.1.16	GNU Project shell	https://www.gnu.org/software/bash/; RRID:SCR_021268
Python 3.10	Python Programming Language	https://www.python.org/: RRID:SCR_008394
R 4.1.2	The R project for statistical computing	https://www.r-project.org/; RRID:SCR_001905
Tidyverse (dplyr 1.1.4, readr 2.1.4, ggplot2 3.4.4)	Wickham et al. 2019	https://www.tidyverse.org/packages/; RRID:SCR_019186
Pandas 2.1.1	Pandas development team, 2020	https://pandas.pydata.org; RRID:SCR_018214
Pandaseq	Masella et al. 2012	https://github.com/neufeld/pandaseq; RRID:SCR_002705
MigMAP	Sangesland et al. 2019	https://github.com/mikessh/migmap
MEGA11 software	Tamura et al. 2011, 2021	https://www.megasoftware.net; RRID:SCR_023017
UCSF ChimeraX	Pettersen et al.2021	https://www.cgl.ucsf.edu/chimerax/; RRID:SCR_015872
Coot 0.9.8	Casanal et al. 2020	http://www2.mrc-lmb.cam.ac.uk/personal/pemsley/coot/; RRID:SCR_014222
Phenix	Afonine et al. 2018	https://phenix-online.org; RRID:SCR_014224
Rosetta	Conway et al. 2014	https://www.rosettacommons.org/home; RRID:SCR_015701
XDSGUI	XDS program package	https://strucbio.biologie.uni-konstanz.de/xdswiki/index.php/XDSGUI; RRID:SCR_015652
Protonate3D tool	Molecular Operating Environment, 2020; Labute, 2009	RRID:SCR_014882
Amber Tools20 package	Case et al. 2020	https://ambermd.org/index.php; RRID:SCR_014230
NpT ensemble	Salomon-Ferrer et al. 2013	N/A
SHAKE algorithm	Miyamoto, 1992	N/A
Berendsen algorithm	Berendsen, 1984	N/A
GetContacts software	This paper	https://getcontacts.github.io/
Other		
Mastercycler PCR machine	Eppendorf	N/A
ELISA readers	BioTek	N/A
5 laser ARIA II	BD Biosciences	RRID:SCR_018091
5 laser LSR Fortessa	BD Biosciences	RRID:SCR_019601
Qubit	Thermo Fisher	RRID:SCR_018095
MiSeq	Illumina	RRID:SCR_016379
Nextseq2000	Illumina	RRID:SCR_023614
Tapestation	Agilent	RRID:SCR_019394
5 Laser Aria SORP	BD Biosciences	N/A
Superdex 200 10/300 Column	GE Healthcare	Cat#17517501
Superose 6 10/300 Column	GE Healthcare	Cat#17517201
96 Well glass bottom plate with high performance #1.5 cover glass	CellVis	Cat#P96-1.5P
CellDiscoverer7	Zeiss	N/A
Tangential flow filtration system	Cytiva	N/A
ViewDrop II seals	SPT LabTech	Cat#4150-05600
*Erythrina cristagalli* Gel-ECA-Immobilized Lectin	EY Laboratories	N/A
AKTA size exclusion chromatography (SEC)	Cytiva	RRID:SCR_023461
IgG Elution Buffer	Pierce	Cat#21009
Cu grids	EMS	Cat#CF300-Cu-UL
Vitrobot	Thermo Fisher	N/A
Glacios-2 cryoEM	Thermo Fisher	N/A
Beam line 24-ID-E	Advanced Photon Source	N/A
BLItz System (Octet N1)	Fortebio	RRID:SCR_023267
Streptavidin biosensors (SA)	Sartorious	Cat#18-5019
Prolab Isopro RMH 3000, 5P75-5P76 mice diet	LabDiet	https://www.labdiet.com/product/detail/5p76-prolab-isopro-rmh-3000

## Highlights

- Natural HA affinity for human bnAb precursors is transduced into vaccine nanoparticles
- A humanized mouse is engineered to contain human bnAb precursor frequency
- Vaccine protection against Group 1 + Group 2 IAV is generated by stem bnAb elicitation
- The antibodies undergo a single amino acid ‘switch’ to accommodate IAV glycan diversity
