# Supplementary material for: Eliciting a single amino acid change by vaccination generates antibody protection against group 1 and group 2 influenza A viruses
Source: Immunity. Author manuscript; Available in PMC 2024 May 16. (PMC11096021; doi:10.1016/j.immuni.2024.03.022)
Supplement: 1 [file NIHMS1984324-supplement-1.pdf]

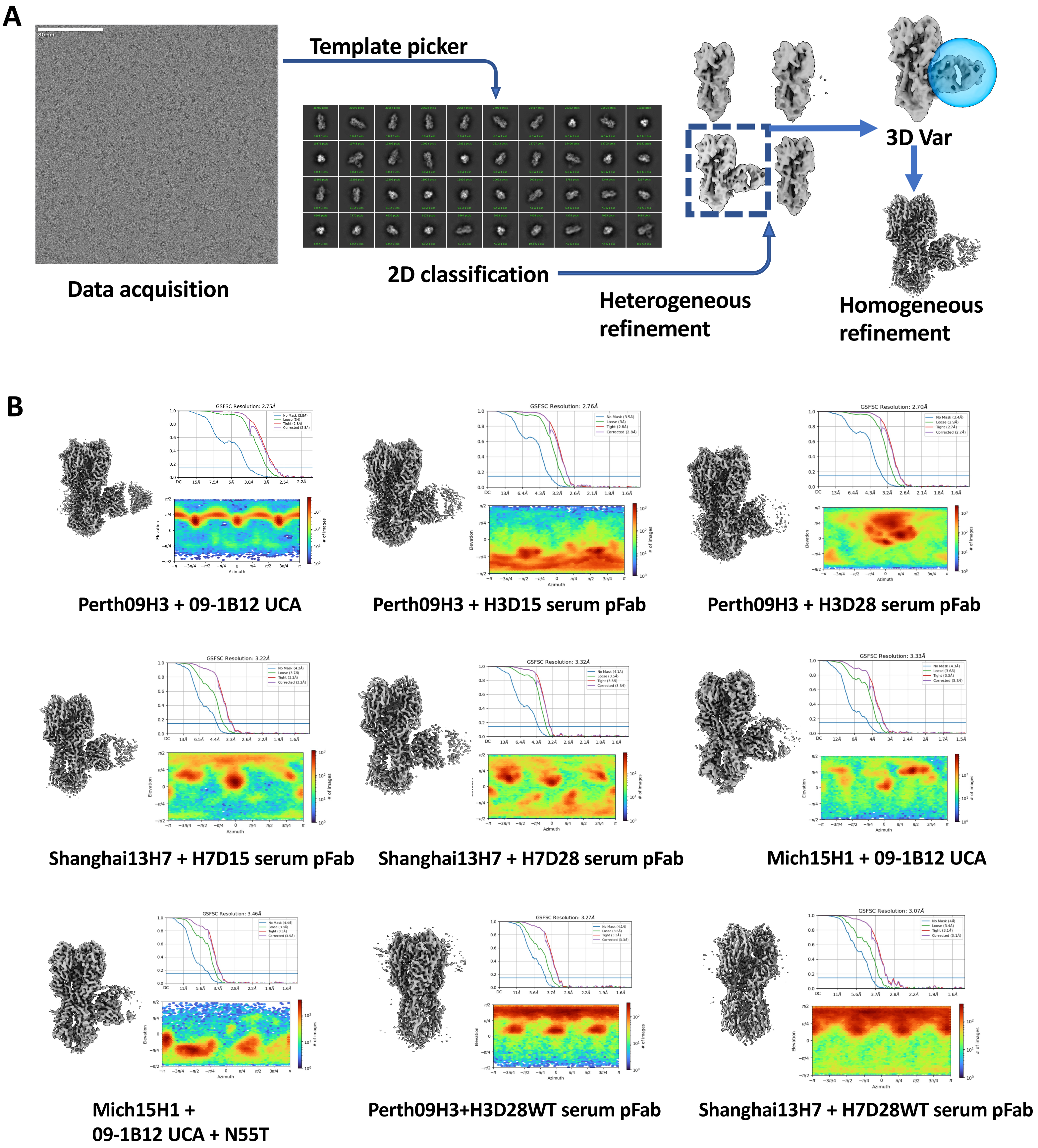

Figure S1. CryoEM data processing and model reconstruction (related to Figure 1B,C, 5D,E,G-I, 6A-F). (A) A general workflow of data processing for HA complexed with monoclonal or polyclonal Fabs. (B**)** Fourier Shell Correlation and angular sampling for cryoEM maps in this manuscript.

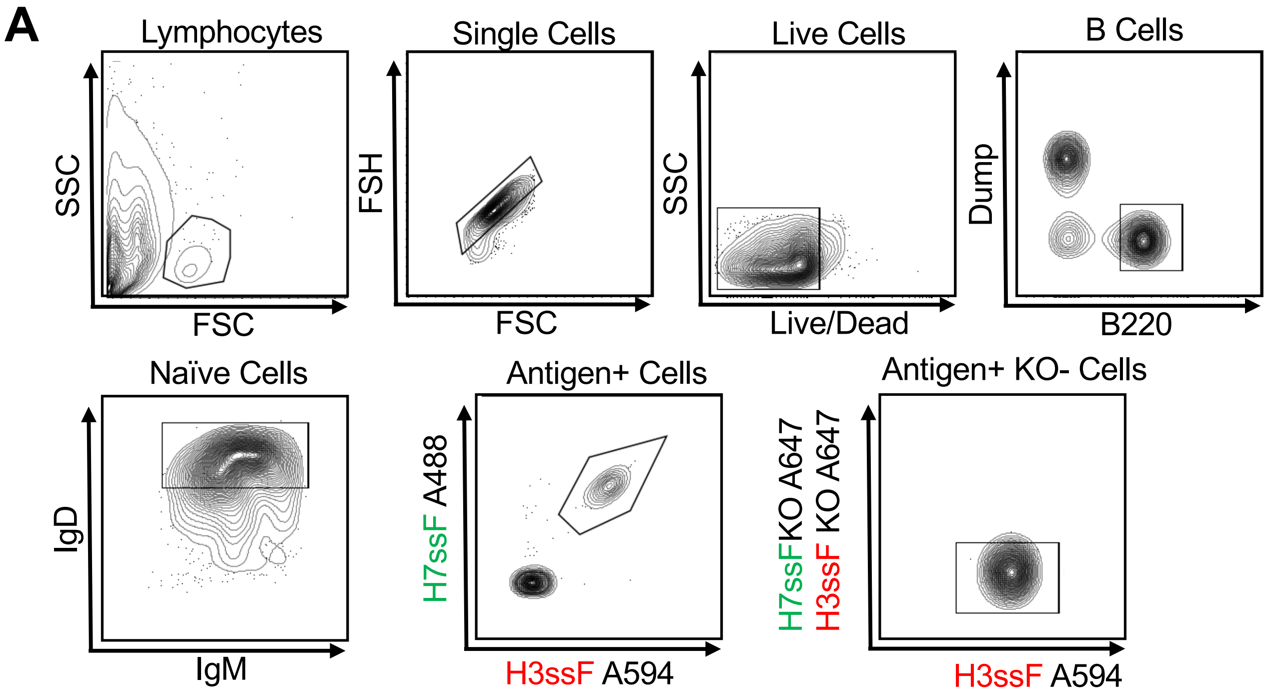

B

	% of B220+ cells
IGHV - 09-1B12-UCA	38.14
IGHK - 09-1B12-UCA	38.56
paired	11.02
IGLV1 - 09-1B12-UCA	17.8
IGLV2 - 09-1B12-UCA	3.81
IGLV3 - 09-1B12-UCA	1.69

C

	% H3+/H7+ cells
IGHV - 09-1B12-UCA	99.19
IGHK - 09-1B12-UCA	83.47
paired	83.47
IGLV1 - 09-1B12-UCA	0.4
IGLV2 - 09-1B12-UCA	0.4
IGLV3 - 09-1B12-UCA	0

Figure S2. 09-1B12-UCA B cell frequency and antigenicity in the KI mice ($H^{09\text{-}1B12\text{-}UCA/WT}$, $\kappa^{09\text{-}1B12\text{-}UCAWT}$) (Related to Figure 2A-D). (A) Flow cytometry gating strategy for sorting and sequencing naïve B cells from 09-1B12-UCA KI mice generated with CRISPR-Cas9. (B) Table showing frequency (%) of amplified human 09-1B12-UCA heavy chain (IGHV), human 09-1B12-UCA light chain (IGKV) and paired human heavy and light chain KI sequences from 10x sequencing of naïve B cells and antigen^{+} B cells (H3ssF^{+}/H7ssF^{+}/H3ssF-KO^{-}/H7ssF-KO^{-}, central stem epitope KO=N-linked glycan at 45_{HA2}) in 09-1B12-UCA KI mice (one experiment).

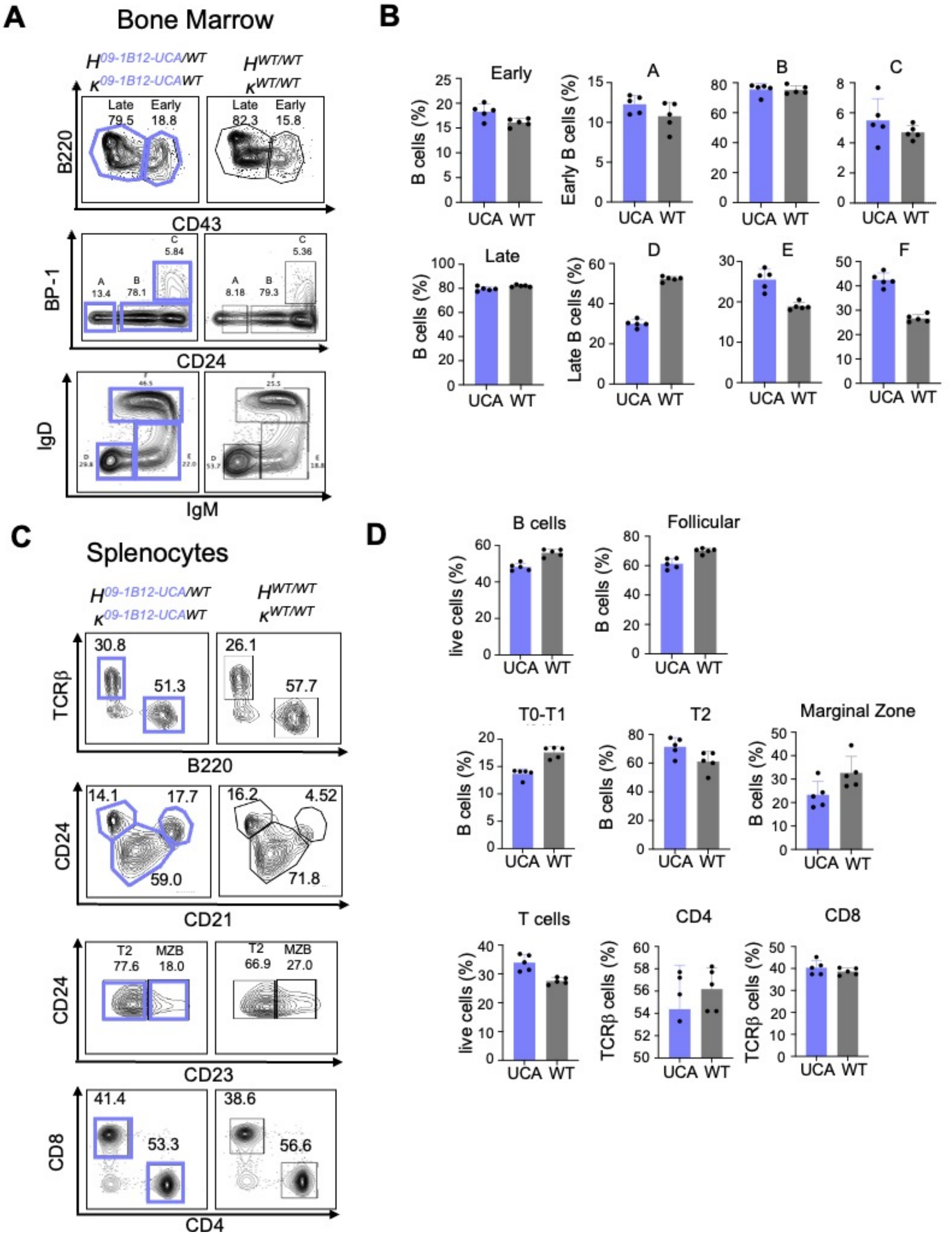

Figure S3. B cell development within the bone marrow and spleen of 09-1B12-UCA KI mice ($H^{09\text{-}1B12\text{-}UCA/WT}$, $\kappa^{09\text{-}1B12\text{-}UCAWT}$) (related to Figure 2A-D). (A) Representative flow cytometry of bone marrow progenitors isolated from 8–10-week-old $H^{09\text{-}1B12\text{-}UCA/WT}$ $\kappa^{09\text{-}1B12\text{-}UCA/WT}$ and WT mice, separated by Hardy classification into early ($B220^{+}/CD43^{+}$) and late ($B220^{+}/CD43^{-}$) B cells (top panel). Middle and bottom panels show early ($BP\text{-}1^{+}/CD24^{+}$) B cell subfractions and late (IgD^{+}/IgM^{+}) subfractions respectively in the bone marrow. (B) Quantification of early and late B cell fractions in bone marrow of $H^{09\text{-}1B12\text{-}UCA/WT}$, $\kappa^{09\text{-}1B12\text{-}UCA\ /WT}$ and WT mice from (A) (n=5 mice per genotype, mean ± SD, one experiment). (C) Representative flow cytometry of splenocytes isolated from 8–10-week-old $H^{09\text{-}1B12\text{-}UCA/WT}$, $\kappa^{09\text{-}1B12\text{-}UCA\ /WT}$ and WT mice and gating strategy applied for the quantification of splenic B cells and their differentiation into Follicular, Transitional (T0, T1, T2) and Marginal Zone B cells. Bottom plots show CD4 and CD8 T cells in spleens of 09-1B12-UCA KI and WT mice. (D) Quantification of B and T cells and different fractions of B cells from sample in (C) (n=5 mice per genotype, mean ± SD, one experiment).

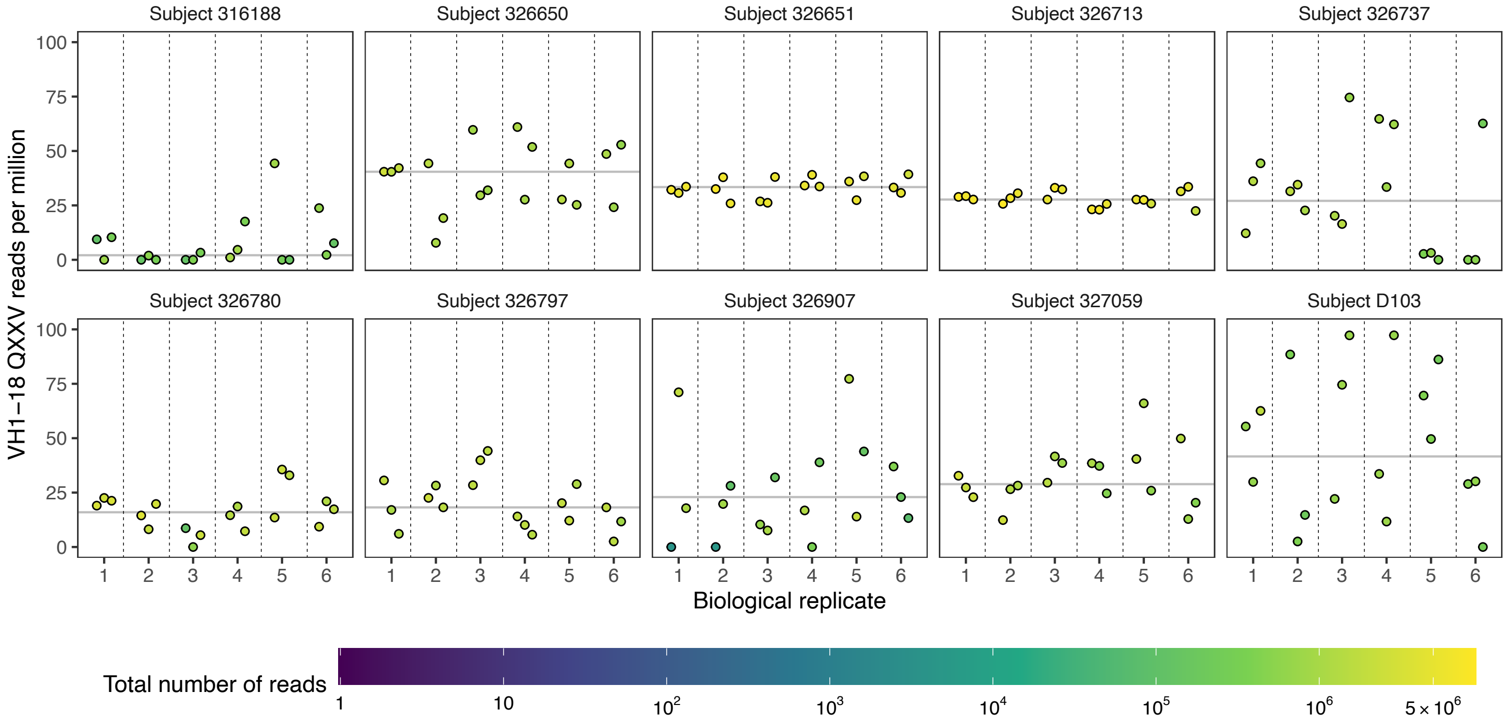

Figure S4. VH1-18 QxxV class bnAb precursors frequency in humans (related to Figure 2E). (A) Frequencies of the bnAb precursors within deep sequenced human IgM antibody repertoires (n=10 subjects with six biological replicates per subject and three technical replicates (three points shown) per biological replicate (1-6 on x-axis) (Briney *et al.*, 2019). The median frequency, excluding replicates with fewer than 100,000 total reads, is shown by the gray line (median = 27.4 per million reads with interquartile range = 22.1 per million reads).

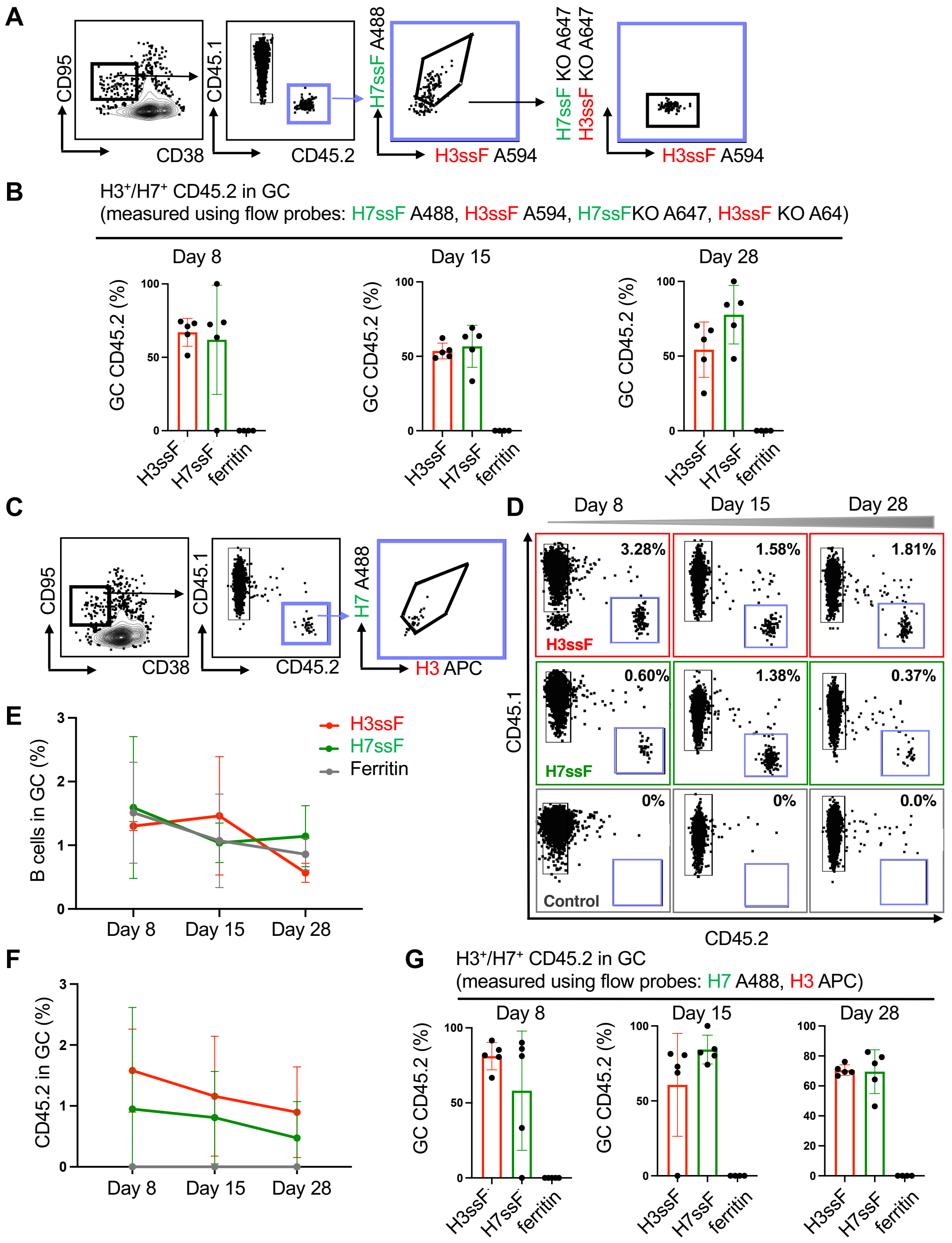

Figure S5. Flow cytometry on germinal center reactions following single immunization with H3ssF, H7ssF or ferritin alone (related to Figure 3A-E). (A) Representative flow cytometry gating for germinal center responses when HA nanoparticle probes are deployed to detect epitope specificity (H3ssF-A594, H7ssF-A488, H7ssF-KO-A647 + H3ssF-KO-A647; central stem epitope KO=N-linked glycan at 45_{HA2}). (B) Percentage of H3ssF$^+$/H7ssF$^+$/ H3ssF-KO$^-$/H7ssF-KO$^-$ reactive CD45.2 B cells that are within the GCs at Days 8, 15, and 28 post immunization with H3ssF, H7ssF or ferritin control (n=5 mice per immunization regimen). (C-G) Germinal center responses when H3 trimer and H7 trimer probes (instead of nanoparticle probes) are deployed to detect epitope specificity. (C) Representative flow cytometry gating for H7$^+$/H3$^+$ cross-reactivity. (D) Representative flow plots of CD45.2 B cells being recruited to GCs at days 8, 15, and 28 post-vaccination. (E) The percentage of GC B cells in the CD45.1 host was quantified at each time point (n=5 mice per group, mean ± SD). (F) The percentage of CD45.2 B cells within the host GCs each time point (n=5 mice per group, mean ± SD).(G) Percentage of H3$^+$/H7$^+$ cross-reactive CD45.2 B cells that are within the GCs at Days 8, 15, and 28 post immunization with H3ssF, H7ssF or ferritin control (n=5 mice per immunogen, one experiment).

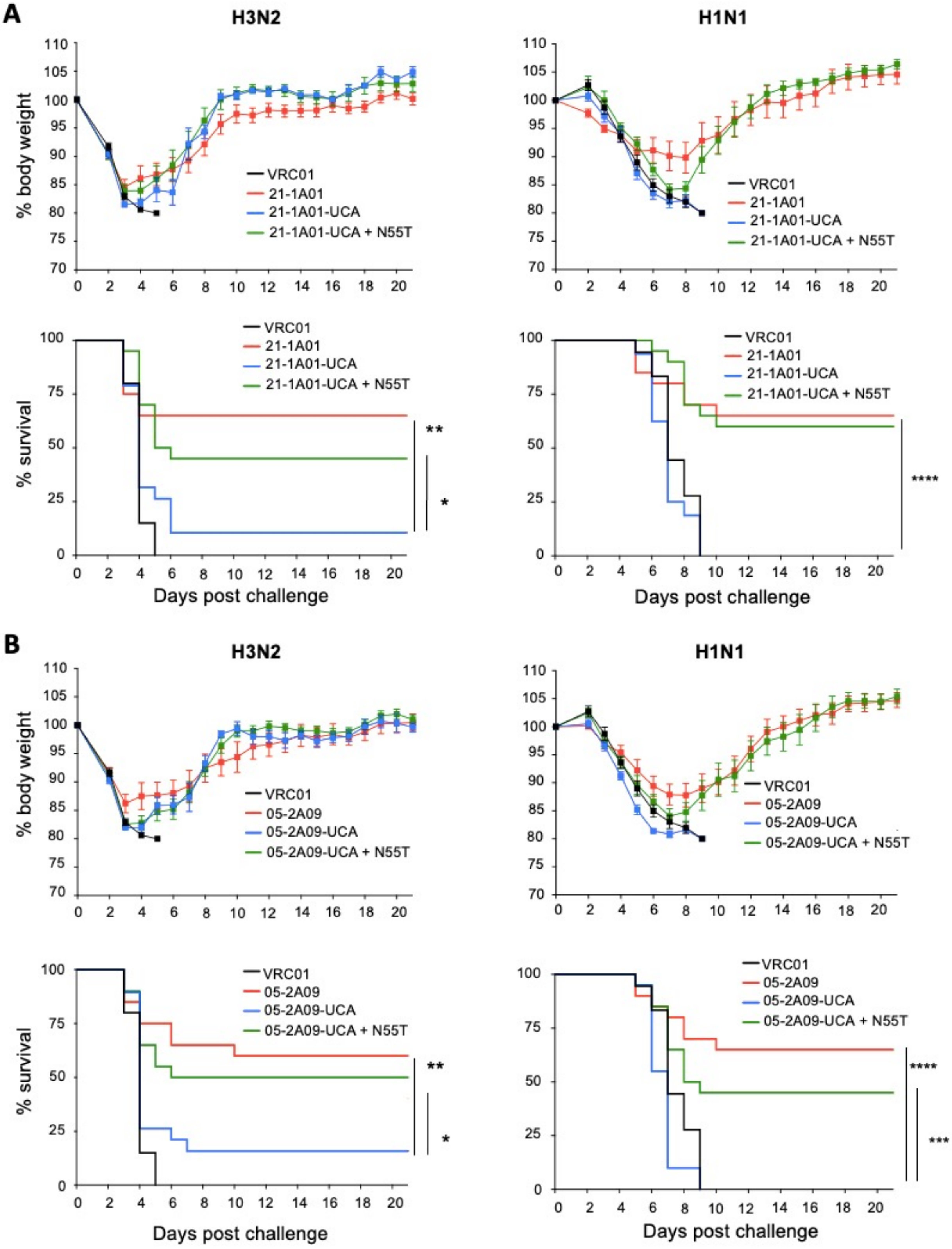

Figure S6. N55T enables cross-group protection by multiple members of the VH1-18 QxxV bnAb class (related to Figure 6A-D). (A) Weight loss and survival from lethal H3N2 virus challenge (10^8 $TCID_{50}$/ml X-31) and H1N1 virus challenge [10^4 $TCID_{50}$/ml maA/Cal/09 (Fink *et al.*, 2018; Ursin *et al.*, 2022)] following passive transfer of 5 mg/kg 21-1A01-UCA, 21-1A01-UCA + N55T, 21-1A01, or VRC01 as the isotype control [n=20 mice per group, ****P<0.0001, **P<0.01, *P<0.05 (Mantel-Cox test of survivorship); one experiment]. (B) Weight loss and survival from lethal H3N2 virus challenge (10^8 $TCID_{50}$/ml X-31) and H1N1 virus challenge (10^4 $TCID_{50}$/ml maA/Cal/09) following passive transfer of 5 mg/kg 05-2A09-UCA, 05-2A09-UCA + N55T, 05-2A09, or VRC01 as the isotype control [n=20 mice per group, ****P<0.0001, ***P<0.001, **P<0.01, *P<0.05 (Mantel-Cox test of survivorship); one experiment].

Table S1. CryoEM data collection, processing and model building statistics, related to Figure 1,5,7 and S1.

Map	Perth09H3+UCA6 FAb	Perth09H3 + H3D15 pFab	Perth09H3 + H3D28 pFab	Shanghai13H7 + H7D15 pFab	Shanghai13H7 + H7D28 pFab	Mich15H1 + 091B12 Fab	Mich15H1 + UCA_N55T Fab
EMDB	EMD-42528	EMD-42531	EMD-42532	EMD-42533	EMD-42534	EMD-42529	EMD-42530
Data collection							
Microscope				TFS Glacios			
Voltage (kV)				200			
Detector				TFS Falcon 4			
Recording mode				Counting			
Nominal magnification				190,000x			
Movie micrograph pixelsize (Å)				0.725			
Number of frames (Falcon 4 EER fractions)				40			
Total dose ($e^-/Å^2$)	50.21	50.36	50.36	49.19	46.87	49.19	49.22
Defocus range (µm)				-0.7 to -1.5			
EM data processing							
Number of movie micrographs	5,565	4,457	4,238	4,553	8,990	4,766	4,805
Particles used for reconstruction	469,712	382,032	477,373	167,583	183,753	89,540	77,014
Symmetry				C1			
Map pixel size				0.725			
Map resolution (FSC 0.143; Å)	2.8	2.8	2.7	3.2	3.3	3.3	3.5
Map sharpening B-factor ($Å^2$)	-72.9	-77.1	-76.7	-92.3	-92.7	-73.4	-86.6
Structure building and validation							
Composition							
Atoms	13696	13046	13046	12707	13329	13663	13615
Residues (protein)	1690	1693	1693	1684	1684	1694	1693
Residues (ligands)	NAG:27	NAG: 24	NAG: 24	NAG:15	NAG:15	NAG:18	NAG:17
MolProbity score	1.05	1.32	1.25	1.38	1.34	1.32	1.15
Clashscore	2.34	3.58	3.38	3.81	4.21	2.83	3.59
Map correlation coefficient (mask)	0.84	0.79	0.84	0.76	0.78	0.80	0.81
EMRinger score							
d FSC model (0.5; Å)	2.9	3.0	2.9	3.6	3.5	3.6	3.9
RMSD from ideal							
Bond length (Å)	0.005	0.005	0.006	0.006	0.006	0.006	0.007
Bond angles (˚)	0.966	0.901	1.061	1.120	1.083	1.073	1.104
Ramachandran plot							
Favored (%)	97.85	97.02	97.38	96.70	97.30	96.31	98.03
Allowed (%)	2.15	2.98	2.62	3.30	2.70	3.69	1.97
Outliers (%)	0	0	0	0	0	0	0
Side chain rotamer outliers (%)	0	0	0	0	0	0.07	0
Cβ outliers (%)	0	0	0	0	0	0	0
PDB	8UT3	8UT6	8UT7	8UT8	8UT9	8UT4	8UT5

Table S2. Crystallographic statistics of 09-1B12 Fab bound to H3 HA A/Perth/16/2009, related to Figures 1, 4 and 7

	09-1B12 Fab bound to H3 HA A/Perth/16/2009
Data Collection	
Resolution (Å)	50.21 - 4.02
Wavelength (Å)	0.97918
Space Group	P $3_1$21
Unit cell dimensions (a, b, c) (Å)	158.04, 158.04, 417.206
Unit cell angles (α, β, γ) (°)	90, 90, 120
I/σ	7.00 (1.67)
R_{meas} (%)	0.5988 (2.305)
$CC_{1/2}$ (%)	99.5 (65.4)
Completeness (%)	99.69 (98.07)
Number of observed reflections	1045911 (93567)
Number of unique reflections	51070 (4940)
Redundancy	20.5 (18.9)
Refinement	
Resolution (Å)	50.21 - 4.02 (4.164 - 4.021)
Reflections used in refinement	51049 (4939)
Reflections used for R_{free}	2003 (194)
R_{work} (%)	22.98 (29.93)
R_{free} (%)	25.44 (33.87)
Ramachandran favored/allowed (%)	95.44 / 4.34
Ramachandran outliers (%)	0.22
Rmsd bond lengths (Å)	0.005
Rmsd bond angles (°)	0.73
Average B-factor	113.41
PDB ID	8UWA

Table S3: Binding constants (K_D) for UCA-inferred VH1-18 QxxV ± N55T Fabs to group 1 and 2 IAV HAs (H1 = A/Michigan/45/2015, pdmH1 = A/California/07/2009, H5 = A/Indonesia/05/2005, H3 = A/Perth/16/2009; H7 = A/Shanghai/02/2013). Related to Figures 1, 6 and S6.

Fab	Antigen (HA)	K_D (M)	Ka (M-1 s-1)	Ka Error	Kd (s-1)	Kd Error
09-1B12-UCA	H1	0.0004363.3	2.91E+03	1.41E+05	1.27E+00	4.92E-01
	pdmH1	8.58E-04	2.54E+02	8.18E+03	2.18E-01	2.24E-02
	H5	3.28E-03	6.45E+01	7.47E+03	2.12E-01	1.32E-02
	H3	1.24E-07	2.10E+04	2.18E+02	2.60E-03	4.01E-05
	H7	2.68E-06	1.21E+04	9.55E+01	3.26E-02	8.80E-05
05-2A09-UCA	H1	1.60E-03	1.33E+02	3.92E+03	2.12E-01	1.30E-02
	pdmH1	2.00E-05	6.87E+03	8.40E+02	1.37E-01	1.88E-03
	H5	6.11E-04	6.35E+02	2.64E+04	3.87E-01	5.65E-02
	H3	6.66E-07	6.77E+04	5.73E+02	4.51E-02	1.92E-04
	H7	3.73E-06	9.94E+03	8.89E+02	3.71E-02	8.81E-04
05-2D04-UCA	H1	8.42E-05	3.63E+03	2.29E+03	3.05E-01	6.17E-03
	pdmH1	2.12E-03	4.89E+01	4.67E+02	1.04E-02	1.08E-03
	H5	2.58E-02	8.08E+00	7.50E+03	2.06E-01	1.85E-02
	H3	2.19E-08	2.72E+04	1.47E+02	5.96E-04	9.46E-06
	H7	4.10E-07	4.01E+03	9.53E+00	1.64E-03	6.00E-06
a06-1F04-UCA	H1	1.42E-04	1.05E+03	6.95E+02	1.49E-01	1.67E-03
	pdmH1	1.67E-03	1.53E+02	6.12E+03	2.51E-01	1.58E-02
	H5	7.05E-05	5.68E+03	2.31E+04	4.00E-01	5.23E-02
	H3	2.61E-08	2.73E+04	1.10E+02	7.13E-04	7.62E-06
	H7	1.86E-07	8.47E+03	4.64E+01	1.57E-03	1.67E-05
21-1A01-UCA	H1	3.60E-05	1.12E+04	3.76E+03	4.02E-01	9.78E-03
	pdmH1	9.34E-05	3.39E+03	1.26E+04	3.17E-01	2.34E-02
	H5	4.61E-06	4.85E+04	1.42E+03	2.24E-01	2.26E-03
	H3	1.39E-08	6.03E+04	1.75E+02	8.42E-04	5.02E-06
	H7	9.81E-08	4.94E+04	2.04E+02	4.84E-03	2.92E-05
27-1D08-UCA	H1	8.12E-04	4.01E+02	6.13E+03	3.26E-01	1.61E-02
	pdmH1	5.30E-05	1.98E+03	5.32E+03	1.05E-01	6.37E-03
	H5	6.96E-03	4.11E+01	3.97E+04	2.86E-01	9.30E-02
	H3	5.45E-07	1.19E-04	2.33E+01	6.47E-03	1.19E-05
	H7	4.24E-06	1.05E+04	5.25E+02	4.45E-02	7.27E-04
09-1B12-UCA + N55T	H1	8.26E-06	1.53E+04	8.66E+02	1.26E-01	1.73E-03
	pdmH1	1.96E-06	7.52E+04	3.41E+03	1.43E-01	2.96E-03
	H5	1.27E-05	1.51E+04	1.27E+03	1.91E-01	2.77E-03
	H3	7.43E-08	6.20E+03	1.31E+01	4.61E-04	5.59E-06
	H7	6.53E-07	5.44E+03	9.69E+01	3.55E-03	4.91E-05
05-2A09-UCA + N55T	H1	1.83E-05	1.68E+04	2.65E+03	3.06E-01	5.70E-03
	pdmH1	5.91E-07	2.88E+05	1.51E+04	1.72E-01	4.11E-03
	H5	1.98E-06	5.23E+04	8.90E+02	1.04E-01	5.58E-04
	H3	2.25E-07	5.52E+04	2.52E+02	1.24E-02	4.24E-05
	H7	3.94E-07	3.74E+04	1.41E-02	1.47E-02	3.77E-05
05-2D04-UCA + N55T	H1	5.02E-05	2.76E+03	2.30E+02	1.39E-01	5.77E-04
	pdmH1	7.60E-06	1.50E+04	6.38E+02	1.14E-01	1.16E-03
	H5	4.07E-06	8.39E+03	2.01E+02	3.41E-02	2.48E-04
	H3	8.32E-09	2.14E+04	9.58E+01	1.81E-04	1.19E-05
	H7	5.69E-08	1.12E+04	2.66E+01	6.37E-04	6.77E-06
a06-1F04-UCA + N55T	H1	1.96E-06	6.98E+04	7.31E+02	1.37E-01	4.99E-04
	pdmH1	1.44E-06	9.14E+04	1.15E+03	1.40E-01	4.71E-04
	H5	4.91E-06	1.03E+05	2.55E+04	5.06E-01	3.91E-01
	H3	3.24E-08	2.32E+04	1.50E+02	7.55E-04	1.33E-05
	H7	2.93E-07	4.39E+04	4.69E+03	5.76E-01	1.08E-02
21-1A01-UCA + N55T	H1	6.87E-06	2.53E+04	4.57E+02	1.74E-01	7.81E-04
	pdmH1	4.78E-06	4.93E+04	1.61E+03	2.36E-01	2.47E-03
	H5	2.51E-06	6.16E+04	2.04E+03	1.55E-01	1.75E-03
	H3	2.09E-08	1.79E+04	3.63E+01	3.75E-04	5.40E-06
	H7	2.57E-08	1.52E+04	3.89E+01	3.89E-04	7.01E-06
27-1D08-UCA + N55T	H1	1.17E-06	6.45E+04	2.64E+03	7.52E-02	1.18E-03
	pdmH1	4.87E-06	2.21E+04	1.75E+03	1.08E-01	1.63E-03
	H5	9.52E-06	1.21E+06	2.09E+03	1.16E-01	2.47E-03
	H3	4.25E-07	3.82E+03	6.17E+01	1.62E-03	1.97E-05
	H7	6.77E-07	6.95E+04	2.40E+03	4.70E-02	1.01E-03

Table S5. Primers for single cell BCR sequencing, related to STAR Methods

Primer	Sequence
	Single cell forward
P7_09-1B12_HC	GTCTCGTGGGCTCGGAGATGTGTATAAGAGACAGGTTCAGCTGGTGCAGTCTGG
P7_09-1B12_LC	GTCTCGTGGGCTCGGAGATGTGTATAAGAGACAGTGTGTTGACGCAGTCTCCAGG
	Single cell reverse
P5_09-1B12_HC	TCGTCGGCAGCGTCAGATGTGTATAAGAGACAGCCAGACGTCCATAGTGTAGTAGT AGGA
P5_09-1B12_LC	TCGTCGGCAGCGTCAGATGTGTATAAGAGACAGCCTGGCCAAAAGTCCACCGA
